# Non-Psychoactive Phytocannabinoids Inhibit Inflammation-Related Changes of Human Coronary Artery Smooth Muscle and Endothelial Cells

**DOI:** 10.3390/cells12192389

**Published:** 2023-09-30

**Authors:** Elisa Teichmann, Elane Blessing, Burkhard Hinz

**Affiliations:** Institute of Pharmacology and Toxicology, Rostock University Medical Center, Schillingallee 70, 18057 Rostock, Germany; elisa.teichmann@web.de (E.T.); elane.blessing@med.uni-rostock.de (E.B.)

**Keywords:** cannabidiol, tetrahydrocannabivarin, human coronary artery, smooth muscle cells, endothelial cells, vascular cell adhesion molecule-1, nuclear factor κB, histone deacetylases

## Abstract

Atherosclerosis is associated with vascular smooth muscle cell proliferation, chronic vascular inflammation, and leukocyte adhesion. In view of the cardioprotective effects of cannabinoids described in recent years, the present study investigated the impact of the non-psychoactive phytocannabinoids cannabidiol (CBD) and tetrahydrocannabivarin (THCV) on proliferation and migration of human coronary artery smooth muscle cells (HCASMC) and on inflammatory markers in human coronary artery endothelial cells (HCAEC). In HCASMC, CBD and THCV at nontoxic concentrations exhibited inhibitory effects on platelet-derived growth factor-triggered proliferation (CBD) and migration (CBD, THCV). When interleukin (IL)-1β- and lipopolysaccharide (LPS)-stimulated HCAEC were examined, both cannabinoids showed a concentration-dependent decrease in the expression of vascular cell adhesion molecule-1 (VCAM-1), which was mediated independently of classical cannabinoid receptors and was not accompanied by a comparable inhibition of intercellular adhesion molecule-1. Further inhibitor experiments demonstrated that reactive oxygen species, p38 mitogen-activated protein kinase activation, histone deacetylase, and nuclear factor κB (NF-κB) underlie IL-1β- and LPS-induced expression of VCAM-1. In this context, CBD and THCV were shown to inhibit phosphorylation of NF-κB regulators in LPS- but not IL-1β-stimulated HCAEC. Stimulation of HCAEC with IL-1β and LPS was associated with increased adhesion of monocytes, which, however, could not be significantly abolished by CBD and THCV. In summary, the results highlight the potential of the non-psychoactive cannabinoids CBD and THCV to regulate inflammation-related changes in HCASMC and HCAEC. Considering their effect on both cell types studied, further preclinical studies could address the use of CBD and THCV in drug-eluting stents for coronary interventions.

## 1. Introduction

Cardiovascular diseases (CVDs) are the leading cause of death worldwide, with an estimated number of 17.9 million deaths in 2019 [1]. Most CVDs, especially coronary artery disease (CAD), are caused by atherosclerotic lesions in the arterial wall associated with endothelial dysfunction, accumulation of lipids, vascular smooth muscle cell (VSMC) proliferation, and chronic inflammation (for review, see [2,3]). The main treatment strategy for patients with CAD is the implantation of drug-eluting stents (DES) [4]. In particular, DES consisting of a metal scaffold coated with a carrier substance (e.g., a polymer) and an agent with antiproliferative and antimigratory activity against VSMC are used to allow blood flow and prevent restenosis (for review, see [5]). VSMC play an important role in all stages of atherosclerosis and restenosis because they are plastic and switch from a contractile to a synthetic/proliferative phenotype (for review, see [6,7,8]). However, first-generation DES such as the mechanistic Target of Rapamycin (mTOR) inhibitor sirolimus lead to delayed endothelial healing, decreased reendothelialization, and very late stent thrombosis, which carries a high risk of neoatherosclerosis (for review, see [5,9,10]).

Endothelial dysfunction is associated with impaired vascular homeostasis and endothelial activation, leading to a proinflammatory and procoagulant environment (for review, see [11]). Markers of an inflamed state of the endothelium include decreased nitric oxide (NO) bioavailability and increased synthesis of cytokines, chemokines, and adhesion molecules, for example, vascular cell adhesion molecule-1 (VCAM-1) and intercellular adhesion molecule-1 (ICAM-1) (for review, see [9,12]). This increase in VCAM-1 and ICAM-1 expression, mainly induced by proinflammatory cytokines or lipopolysaccharide (LPS), promotes leukocyte recruitment, adhesion, and transmigration into inflamed tissues and ultimately atherosclerosis (for review, see [12,13,14,15]). Targeting VCAM-1 resulted in inhibition of early plaque formation in low-density lipoprotein receptor (*Ldlr^−/−^*) deficient mice and, thus, plays an important role in early atherosclerosis [16]. Knockdown of ICAM-1 in apolipoprotein E (*ApoE^−/−^*)-deficient mice led to reduced vascular lesion size and has been implicated to exert a more important function in the progression of atherosclerosis [17].

In recent years, the potential treatment of atherosclerosis and inflammation in the cardiovascular system with non-psychoactive phytocannabinoids, such as cannabidiol (CBD), has gained considerable interest. Thus, in relation to VSMC, CBD showed anti-proliferative and antimigratory effects in human umbilical artery smooth muscle cells [18]. Moreover, CBD exerted potent anti-inflammatory effects by reducing high glucose-induced inflammation in coronary artery endothelial cells [19] and inducing endothelium-dependent vascular relaxation in mesenteric arteries [20]. CBD also exhibited protective mechanisms against LPS-induced lung injury [21] and diabetic cardiomyopathy by attenuating inflammation, oxidative stress, cell death, and fibrosis [22]. Tetrahydrocannabivarin (THCV), another phytocannabinoid without psychoactive effects, is also of great interest due to its potential medical benefits in obesity and diabetes [23,24]. In addition, both THCV and CBD were able to prevent hepatic ischemia/reperfusion injury by reducing inflammation [25,26].

Although the benefits of non-psychoactive phytocannabinoids have been described in various infection models, the effects of CBD and, in particular, THCV on coronary vascular cells have not been fully investigated. Here, for the first time, CBD and THCV were comparatively studied in human coronary artery smooth muscle cells (HCASMC) and endothelial cells (HCAEC). Thereby, we demonstrate that both phytocannabinoids possess beneficial effects by inhibiting platelet-derived growth factor (PDGF)-induced proliferation (CBD) and migration (CBD, THCV) of HCASMC. Moreover, both phytocannabinoids attenuated proinflammatory VCAM-1 but not ICAM-1 expression in interleukin (IL)-1β- and LPS-stimulated HCAEC without affecting cell viability. Studies were performed on the possible involvement of p38 mitogen-activated protein kinases (MAPK) and nuclear factor κB (NF-κB) in endothelial cannabinoid effects. Overall, the results presented here provide new insights into a potential application of non-psychoactive phytocannabinoids in DES and provide a good starting point for conducting further preclinical studies with the aim of evaluating the investigated test compounds as DES agents.

## 2. Materials and Methods

### 2.1. Materials

Cannabidiol (CBD, #BN0124) was purchased from Biotrend Chemikalien (Cologne, Germany). Tetrahydrocannabivarin (THCV, #T-094), interleukin-1β (IL-1β) human (#SRP3083), lipopolysaccharide (LPS) from *E. coli* O111:B4 (#L2630), capsazepine (#C191), calcein-AM (#17783), and *N*-acetyl-L-cysteine (NAC, #A7250) were bought from Sigma-Aldrich (Taufkirchen, Germany). Platelet-derived growth factor (PDGF)-BB human (#PHG0041) was purchased from Thermo Fisher Scientific (Schwerte, Germany). AM251 (#71670), AM630 (#10006974), SB203580 (#13067), SP600125 (#10010466), PD98059 (#10006726), BAY 11-7082 (#10010266), trichostatin A (TSA, #89730), and sirolimus (#13346) were obtained from Cayman Chemical (Ann Arbor, MI, USA). WST-1 was bought from Roche Diagnostics (Mannheim, Germany). Leupeptin, dithiothreitol (DTT), and Phalloidin-iFluor^TM^ 555 Conjugate were obtained from Biomol (Hamburg, Germany). Aqua ad iniectabilia was acquired from Braun Melsungen (Melsungen, Germany). Aprotinin, bromophenol blue, hydrogen peroxide solution (H_2_O_2_, 30%), p-coumaric acid, luminol, orthovanadate, paraformaldehyde (PFA), phenylmethanesulfonyl fluoride (PMSF), poly-D-lysine hydrobromide (PDL), and Hoechst 33342 were bought from Sigma-Aldrich. Methanol was bought from J.T. Baker (Griesheim, Germany). Acetic acid, dimethyl sulfoxide (DMSO), ethylenediaminetetraacetic acid (EDTA), glycerin, glycine, hydrochloric acid 37% (HCl), Ponceau S, sodium chloride (NaCl), sodium hydroxide (NaOH), sodium dodecyl sulfate (SDS) ultra-pure, Tris ultrapure, and Tris hydrochloride (Tris HCl) were obtained from AppliChem (Darmstadt, Germany). Furthermore, 2-mercaptoethanol and 4-(2-hydroxyethyl)-1-piperazineethanesulfonic acid (HEPES) were acquired from Ferak Berlin (Berlin, Germany). Acrylamide (Rotiphorese^®^ Gel 30; 37.5:1), albumin (IgG-free), ammonium chloride (NH_4_Cl), ammonium peroxydisulphate (APS), crystal violet, *N*,*N*,*N′*,*N′*-tetramethylethylenediamine (TEMED), Triton^®^ X-100, and Tween^®^ 20 were purchased from Carl Roth (Karlsruhe, Germany). VECTASHIELD Antifade Mounting Medium was bought from Biozol Diagnostics Vertrieb (Eching, Germany). Non-fat milk (NFM) powder was purchased from Bio-Rad Laboratories (Munich, Germany). Gibco^TM^ penicillin-streptomycin, Gibco^TM^ trypsin-EDTA, and Gibco^TM^ trypan blue solution (0.4%) were acquired from Thermo Fisher Scientific. Fetal bovine serum (FBS) and Dulbecco’s phosphate-buffered saline (DPBS) were purchased from PAN-Biotech (Aidenach, Germany).

### 2.2. Cell Culture

Human coronary artery smooth muscle cells (HCASMC, #C-12511) and human coronary artery endothelial cells (HCAEC, #C-12221) were purchased from Promocell (Heidelberg, Germany). HCASMC were cultured in Smooth Muscle Cell Growth Medium 2 (#C-22162) supplemented with 5% fetal calf serum (FCS), 0.5 ng/mL epidermal growth factor (EGF) human, 2 ng/mL basic fibroblast growth factor (bFGF) human, and 5 µg/mL insulin human. HCAEC were cultured in Endothelial Cell Growth Medium MV2 (#C-22121) supplemented with 5% FCS, 5 ng/mL EGF human, 10 ng/mL bFGF human, 0.5 ng/mL vascular endothelial growth factor (VEGF) human, 20 ng/mL insulin-like growth factor, 1 µg/mL ascorbic acid, and 0.2 µg/mL hydrocortisone. All media and supplements were purchased from Promocell. Both media were additionally supplemented with 100 U/mL penicillin and 100 μg/mL streptomycin. The resulting media composition is in the following referred to as “complete growth medium”. Cells were cultured in a humidified atmosphere at 37 °C and 5% CO_2_.

Unless otherwise indicated, all cell experiments were performed with test compounds in medium containing 2% FCS, 100 U/mL penicillin, and 100 μg/mL streptomycin, but no other additives, hereafter referred to accordingly as “reduced medium”. Cells were washed with DPBS before treatment. Test compounds were dissolved in ethanol (CBD, THCV), DMSO (sirolimus, AM251, AM630, capsazepine, SB203580, SP600125, PD98059, BAY 11-7082, TSA), aqua ad iniectabilia (IL-1β, LPS), 0.1 M acetic acid/0.1% (*w*/*v*) albumin (PDGF-BB), or DPBS (NAC). Final concentration of solvents in incubation media of cells treated with test compound and vehicle varied from experiment to experiment but in no case exceeded 0.033% (*v*/*v*) for ethanol, 0.2% (*v*/*v*) for DMSO, 0.1% (*v*/*v*) for aqua ad iniectabilia, 0.025% (*v*/*v*) for 0.1 M acetic acid/0.1% (*w*/*v*) albumin, and 1% for DPBS. Vehicle-treated cells contained the same concentrations and amounts of solvents as compound-treated cells.

The human monocytic cell line THP-1 (ACC16, RRID:CVCL_0006) was purchased from the German Collection of Microorganisms and Cell Cultures (DSMZ; Braunschweig, Germany). THP-1 cells were cultured in RPMI 1640 with L-Glutamine (#BE12-702F, Lonza Group, Basel, Switzerland) supplemented with 10% FBS, 100 U/mL penicillin, and 100 μg/mL streptomycin. Cells were cultured in a humidified atmosphere at 37 °C and 5% CO_2_.

### 2.3. Cellular Viability Assays

Metabolic activity was measured by the WST-1 assay, and cell number was assessed by crystal violet staining. For both assays, cells were seeded in 96-well plates at a density of 5000 cells/well and grown for 24 h in complete growth medium. After washing with DPBS, HCASMC were maintained in reduced medium for an additional 24 h and then stimulated for 144 h with test compounds in reduced medium. HCAEC were maintained in reduced medium and directly stimulated with test substances for 24 h. 

Water-soluble tetrazolium salt WST-1 (4-[3-(4-iodophenyl)-2-(4-nitrophenyl)-2H-5-tetrazolio]-1,3-benzenedisulfonate) was used to measure the metabolic activity of the cells. WST-1 is cleaved to a soluble formazan dye using NAD(P)H from the tricarboxylic acid cycle at the cell surface, with the quantity of dye correlating directly with the number of metabolically active cells. After the incubation period, WST-1 was added at a final dilution of 1:10, and the cells were further incubated for 1 h. Afterwards, absorbance was measured at 450 nm/690 nm using a microplate reader. 

For crystal violet staining, cells were fixed overnight with ice-cold absolute ethanol. Then, cells were incubated with 0.1% (*w*/*v*) crystal violet in 10% (*v*/*v*) ethanol for 30 min followed by washing off the excess dye. Finally, the staining was dissolved with 10% (*v*/*v*) acetic acid and absorbance was measured at 570 nm using a microplate reader.

### 2.4. BrdU Proliferation Assay

HCASMC proliferation was determined using the colorimetric 5-bromo-2′-deoxyuridine (BrdU) cell proliferation ELISA from Roche Diagnostics (Mannheim, Germany). To this end, HCASMC were seeded at a density of 5000 cells/well in 96-well plates and grown for 24 h in complete growth medium. Then, cells were maintained in reduced medium for an additional 24 h and subsequently stimulated with test substances for 144 h. BrdU reagent was added 24 h prior to analysis. The assay was performed according to the manufacturer’s instructions.

### 2.5. Scratch Wound Assay

HCASMC migration was determined by a scratch wound assay. Cells were seeded in 24-well plates at a density of 50,000 cells/well and grown in complete growth medium until 100% confluence was reached. Then, cells were maintained in reduced medium (0.5% FCS-containing smooth muscle cell medium supplemented with 100 U/mL penicillin and 100 μg/mL streptomycin) for 24 h. Subsequently, a scratch was made in the cell layer in a straight line, and the debris was rinsed away with DPBS. Cells were stimulated with test compounds, and the scratch wound was observed after 0 h and 24 h by imaging with the AxioVert.A1 from Carl Zeiss (Oberkochen, Germany). The wound area at time 0 h and 24 h was analyzed using Zen2 software (version 2.0.0.0) from Carl Zeiss. The wound closure was calculated as follows: [(wound area after 0 h − wound area after 24 h)/wound area after 0 h] × 100%.

After finishing the scratch wound assay, cells were fixed overnight with ice-cold absolute ethanol before incubation with 0.1% (*w*/*v*) crystal violet in 10% (*v*/*v*) ethanol for 30 min. Excess dye was washed away with DPBS and scratch wound was imaged using AxioVert.A1 from Carl Zeiss.

### 2.6. Quantitative Reverse Transcriptase Polymerase Chain Reaction (qRT-PCR)

HCAEC were seeded at 200,000 cells/well in 6-well plates and grown in complete growth medium for 24 h. After washing with DPBS, HCAEC were maintained in reduced medium and incubated with test compounds for 24 h. Cells were harvested by collecting the cell supernatants and washing with warm DPBS. Cells were then separated with trypsin-EDTA, collected with the cell supernatants, and centrifuged at 200× *g*, 4 °C for 5 min. The cell pellets were again washed with DPBS and centrifuged at 250× *g*, 4 °C for 5 min. Total RNA was isolated from resulting cell pellets using the RNeasy Mini Kit from Qiagen (Hilden, Germany) according to the manufacturer’s instructions. Total RNA concentrations were determined using the NanoDrop™ OneC Microvolume UV-Vis spectrophotometer (Thermo Fisher Scientific).

For quantification of VCAM-1 and ICAM-1 mRNA expression, Applied Biosystems TaqMan^®^ Gene Expression Assay (VCAM-1: Hs01003372_m1; ICAM-1: Hs00164932_m1; both FAM-MGB) and Applied Biosystems^®^ TaqMan^®^ RNA-to-CT™ 1-Step Kit from Thermo Fisher Scientific were used according to the manufacturer’s instructions. Peptidylprolyl isomerase A (PPIA: Hs999904_m1; VIC-MGB) was used as a housekeeping gene to normalize VCAM-1 and ICAM-1 mRNA levels before comparison with respective vehicle controls. 

### 2.7. Total Cellular Protein Isolation

HCAEC were seeded at a density of 200,000 cells/well in 6-well plates and were grown for 24 h in complete growth medium. After washing with DPBS, HCAEC were maintained in reduced medium and incubated with test compounds.

For the analysis of phospho-IκBα/IκBα, phospho-IKKα/β/IKKβ, and phospho-p38/p38, as well as phospho-HDAC4/5/7, HDAC4, HDAC5, HDAC7, HCAEC were washed with ice-cold DPBS and placed in lysis buffer (2% [*w*/*v*] SDS, 40% [*v*/*v*] aqua ad iniectabilia, 10% [*v*/*v*] glycerol, 50% [*v*/*v*] 125 mM Tris-HCl [pH 6.8]). Cells were incubated at 95 °C for 10 min under shaking followed by centrifugation at 20,817× *g*, 4 °C for 5 min. The resulting supernatant was collected and stored for further protein analysis. 

For analysis of VCAM-1 and ICAM-1, cell supernatant was collected, and HCAEC were washed with warm DPBS. Cells were then detached with trypsin-EDTA, collected with the cell supernatant, and centrifuged at 200× *g*, 4 °C for 5 min. Again, cell pellets were washed with DPBS and centrifuged at 250× *g*, 4 °C for 5 min before adding lysis buffer (50 mM HEPES, 1 mM EDTA, 150 mM NaCl, 1% [*v*/*v*] Triton^®^ X-100, 10% [*v*/*v*] glycerol, 1 mM orthovanadate, 1 mM PMSF, 10 µg/mL aprotinin, 1 µg/mL leupeptin). After overnight incubation at −20 °C, the tubes were vortexed and centrifuged at 20,817× *g*, 4 °C for 5 min. The resulting supernatant was collected and stored at −20 °C for further protein analysis. 

Protein concentrations were determined using the Pierce™ BCA Protein Assay Kit (Thermo Fisher Scientific) according to the manufacturer’s instructions.

### 2.8. Nuclear Protein Isolation

HCAEC were seeded, treated, and harvested as described for cellular protein isolation and analysis of VCAM-1 and ICAM-1. To isolate the nuclear fraction and to obtain the cytosolic fraction, NE-PER™ Nuclear and Cytoplasmic Extraction Reagents (Thermo Fisher Scientific) were used according to the manufacturer’s instructions. 

### 2.9. Western Blot Analysis

Equal quantities of denatured protein were separated on 8%, 10%, or 15% SDS-polyacrylamide gels. To determine the molecular weight of the bands, the Precision Plus Protein™ Dual Color Standard from Bio-Rad Laboratories was used. After separation, proteins were transferred to nitrocellulose membranes and subsequently membranes were blocked with 5% (*w*/*v*) NFM in Tris-buffered saline with 0.1% (*v*/*v*) Tween^®^ 20 (TBS-T buffer) for 1 h. The membranes were then rinsed with a mixture of NFM and TBS-T buffer. After washing, membranes were incubated with primary antibodies overnight at 4 °C. Primary antibodies were diluted in 1% (*w*/*v*) NFM, 5% (*w*/*v*) NFM, or 5% (*w*/*v*) albumin in TBS-T buffer according to the manufacturer’s instructions. VCAM-1 (#sc-13160, RRID:AB_626846) and ICAM-1 antibodies (#sc-8439, RRID:AB_627123) were bought from Santa Cruz Biotechnology (Heidelberg, Germany). Phospho-IκBα (Ser32/36) (#9246, RRID:AB_2267145), IκBα (#4814, RRID:AB_390781), phospho-IKKα/β (Ser176/180) (#2697, RRID:AB_2079382), IKKβ (#8943, RRID:AB_11024092), phospho-NF-κB p65 (Ser536) (#3033, RRID:AB_331284), phospho-p38 MAPK (Thr180/Tyr182) (#9211, RRID:AB_331641), p38 MAPK (#9212, RRID:AB_330713), phospho-HDAC4 (Ser246)/HDAC5 (Ser259)/HDAC7 (Ser155) (#3443, RRID:AB_2118723), HDAC4 (#15164, RRID:AB_2798733), HDAC5 (#20458, RRID:AB_2713973), and HDAC7 (#33418, RRID:AB_2716756) antibodies were acquired from Cell Signaling Technology (Frankfurt/Main, Germany). Lamin B1 antibody (#ab16048, RRID: AB_443298) was bought from Abcam (Berlin, Germany). β-actin (#A5441, RRID:AB_476744) and GAPDH (#G9545, RRID: AB_796208) antibodies were obtained from Sigma-Aldrich. After incubation with primary antibodies, membranes were washed with TBS-T buffer and incubated with horseradish peroxidase-linked secondary antibodies (anti-mouse antibody, #7076, RRID:AB_330924; anti-rabbit antibody, #7074, RRID:AB_2099233 from Cell Signaling Technology) in 1% (*w*/*v*) NFM in TBS-T buffer for 1 h at room temperature. Binding of antibodies was visualized by chemiluminescence detection using a substrate solution (100 mM Tris-HCl pH 8.5, 1.25 mM luminol, 200 µM p-coumaric acid, 0.09% [*v*/*v*] H_2_O_2_), and signals were detected using the ChemiDoc XRS gel documentation system from Bio-Rad Laboratories (Munich, Germany).

Signal intensity quantification was performed with Quantity One 1-D analysis software (version 4.6.8, Bio-Rad Laboratories). Signals of the target proteins were normalized to signals of the housekeeping proteins (β-actin for total cellular lysates except for analysis of HDAC, GAPDH for cytoplasmic fractions, and lamin B1 for nuclear fractions and analysis of HDAC). The signals from phosphorylated IκBα, IKKα/β, p38, and the corresponding unphosphorylated proteins were first normalized to β-actin. Then, the ratio of protein expression levels of phosphorylated to unphosphorylated proteins was calculated, which is referred to as "activation of phosphorylation" in the figures. Finally, all protein levels were calculated as percentages relative to the corresponding vehicle control.

### 2.10. Monocyte Adhesion Assay

HCAEC were seeded in PDL-coated 8-well slides at a density of 50,000 cells/well and grown for 24 h in complete growth medium. After washing with DPBS, HCAEC were maintained in reduced medium and incubated with test substances for 24 h. Before starting the adhesion assay, THP-1 cells were labeled with 5 µM calcein-AM in serum-free RPMI-1640 medium for 30 min. THP-1 cells were then washed with DPBS and resuspended in reduced endothelial cell medium. Thereafter, HCAEC were washed with DPBS and 100,000 labeled THP-1 cells/well were added to the endothelial cells. The THP-1 cells were allowed to adhere for 30 min before the nonadherent cells were removed by washing with DPBS. Next, the cells were fixed with 4% (*w*/*v*) PFA for 10 min and neutralized with 50 mM NH_4_Cl for an additional 5 min. Subsequently, the cells were permeabilized by adding DPBS with 0.3% (*v*/*v*) Triton^®^ X-100 for 15 min. After washing with DPBS, the free binding sites were blocked with 0.5% (*w*/*v*) albumin in DPBS containing 0.1% (*v*/*v*) Tween^®^ 20 (PBS-T buffer) for 5 min. The fixed cells were washed with PBS-T and then incubated with Hoechst 33342 fluorescent dye and Phalloidin-iFluor^TM^ 555 Conjugate for 1 h. Cells were then incubated with PBS-T buffer. Finally, the cells were washed again with DPBS and the fluorescence was preserved with VECTASHIELD Antifade Mounting Medium. Fluorescence images were obtained using the AxioScope.A1 from Carl Zeiss (Oberkochen, Germany). To quantify monocyte adhesion, the number of cells stained with calcein-AM was related to the number of cells stained with Hoechst 33342.

### 2.11. Statistics

Statistical analyses were performed using GraphPad Prism 9.1.0 or a later version (GraphPad Software, San Diego, CA, USA). Comparison between two groups was performed using the unpaired, two-tailed Student’s *t* test. To compare more than two groups, a one-way ANOVA with a Dunnett post hoc test was performed when all conditions were compared with the vehicle group and a one-way ANOVA with a Bonferroni post hoc test was performed when selected groups were compared.

## 3. Results

### 3.1. Nontoxic Concentrations of CBD and THCV Inhibit PDGF-Induced Migration of HCASMC with Additional Inhibition of PDGF-Induced Proliferation by CBD

VSMC are able to switch from a contractile to a synthetic/proliferative phenotype (for review, see [8]). Increased smooth muscle cell proliferation and migration occurs as a vascular response resulting from injury caused by a percutaneous procedure. Accordingly, the therapeutic approach here involves the application of DES agents that exert an inhibitory effect on VSMC proliferation and migration [7].

**Figure 1:**
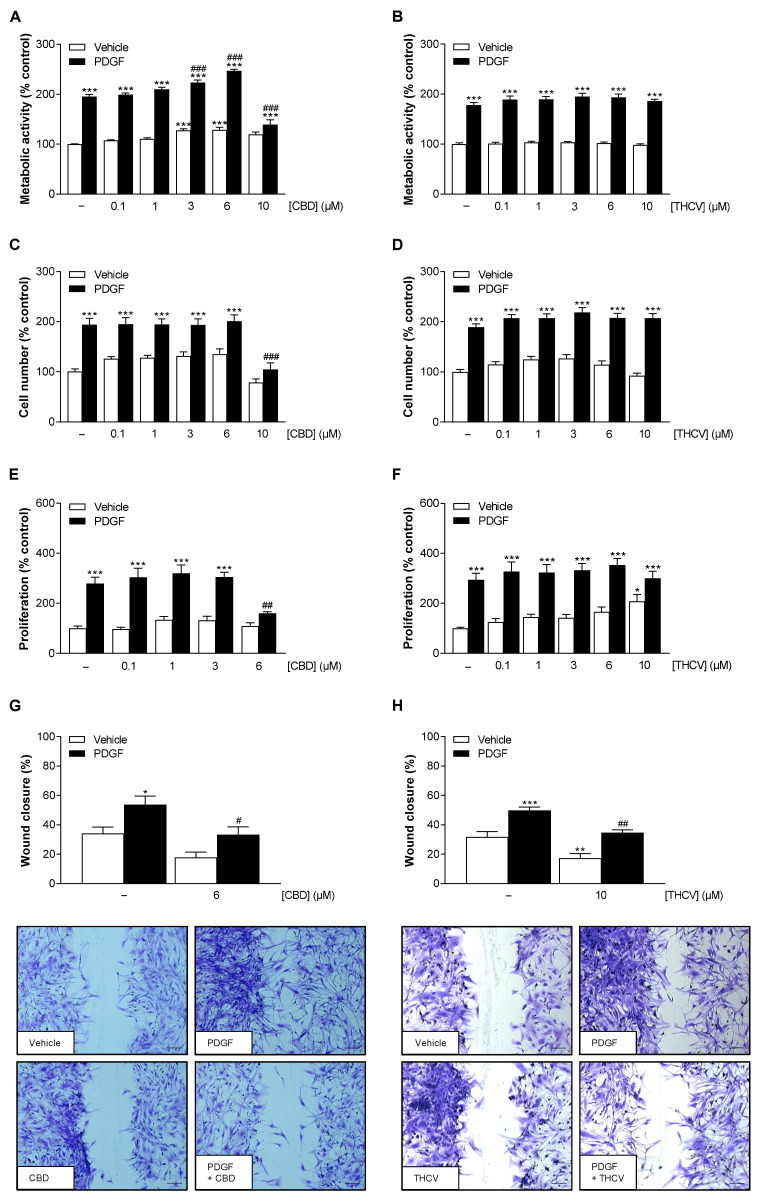
Effect of CBD and THCV on PDGF-induced metabolic activity, cell number, proliferation, and migration of HCASMC. To analyze metabolic activity (**A**,**B**), cell number (**C**,**D**), and proliferation (**E**,**F**), HCASMC were incubated with 25 ng/mL PDGF or its vehicle and increasing concentrations of CBD or THCV or its vehicle for 144 h. Thereafter, metabolic activity was determined by WST-1 colorimetric assay, cell number by crystal violet staining, and proliferation by BrdU incorporation assay. For analysis of migration (**G**,**H**), a scratch wound was made in confluent HCASMC. Cells were then incubated with 25 ng/mL PDGF or its vehicle and increasing concentrations of CBD and THCV or its vehicle. Scratch wounds were analyzed after 0 h and 24 h of incubation. Representative images of crystal violet staining (**G**,**H**) show the scratch wound after 24 h (scale bar, 200 µm). Vehicle-treated cells were used as control (100%), except for migration experiments, where the wound area after 24 h was set in relation to the wound area after 0 h. Data are presented as means ± SEM of *n* = 9–12 (3 independent experiments, (**A**–**D**)), *n* = 9 (3 independent experiments, (**E**,**F**)), and *n* = 6–7 (3–4 independent experiments, (**G**,**H**)). * *p* ≤ 0.05, ** *p* ≤ 0.01, *** *p* ≤ 0.001 vs. vehicle control; # *p* ≤ 0.05, ## *p* ≤ 0.01, ### *p* ≤ 0.001 vs. PDGF-stimulated cells; one-way ANOVA plus Bonferroni post hoc test.

To investigate whether CBD and THCV affect HCASMC viability under basal and PDGF-induced conditions, metabolic activity and cell number were first determined. To this end, HCASMC were treated with PDGF in the absence or presence of increasing concentrations of CBD or THCV for 144 h. Thereby, significant increases in metabolic activity and cell number of HCASMC were registered by PDGF compared to baseline conditions (Figure 1A–D). CBD showed a concentration-dependent increase in basal and PDGF-stimulated metabolic activity at concentrations up to 6 µM (Figure 1A), with no change in cell number under either condition (Figure 1C). However, a significant decrease in both parameters was caused by 10 µM CBD, so this concentration was not considered for further studies. On the other hand, THCV showed no significant effects on basal and PDGF-enhanced metabolic activity and cell number of HCASMC in the entire concentration range up to and including 10 µM (Figure 1B,D). 

Next, the extent to which nontoxic CBD and THCV concentrations attenuate PDGF-stimulated HCASMC proliferation and migration in terms of a possible restenosis-preventing effect was investigated. To examine the proliferation of HCASMC, cells were treated with test compounds for 144 h and then analyzed by BrdU incorporation assay. HCASMC migration was determined after 0 h and 24 h of incubation by a scratch wound assay. PDGF treatment of HCASMC significantly upregulated HCASMC proliferation and migration compared with vehicle control (Figure 1E–H). CBD significantly inhibited PDGF-induced HCASMC proliferation at 6 µM, whereas lower concentrations showed no antiproliferative effect (Figure 1E). Moreover, HCASMC migration was significantly reduced by 6 µM CBD (Figure 1G). Unlike CBD, THCV displayed no effect on PDGF-induced HCASMC proliferation (Figure 1F), though HCASMC migration was significantly inhibited by 10 µM THCV (Figure 1H).

### 3.2. CBD and THCV Do Not Impair the Viability of HCAEC at Low Concentrations

Following stent implantation, DES reendothelialization plays a critical role in preventing restenosis. Therefore, drugs used in DES should promote wound healing and endothelial viability [9]. 

**Figure 2:**
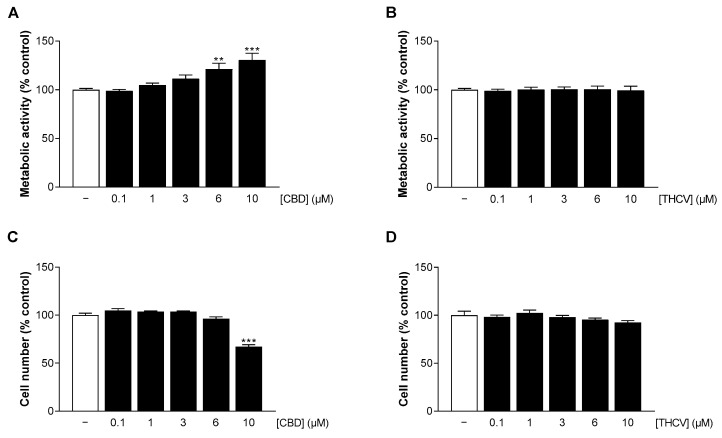
Effect of CBD and THCV on metabolic activity and cell number of HCAEC. HCAEC were incubated with increasing concentrations of CBD (**A**,**C**) or THCV (**B**,**D**) or with vehicle for 24 h. Thereafter, metabolic activity was determined by WST-1 assay (**A**,**B**) and cell number by crystal violet staining (**C**,**D**). Vehicle-treated cells were used as controls (100%). Data are presented as means ± SEM of *n* = 9 (3 independent experiments). ** *p* ≤ 0.01, *** *p* ≤ 0.001 vs. vehicle control; one-way ANOVA plus Dunnett post hoc test.

To test the impact of CBD and THCV on HCAEC, metabolic activity and cell number were determined after 24 h of incubation. Here, no significant change in HCAEC metabolic activity and cell number was observed by CBD concentrations up to 3 µM (metabolic activity) and 6 µM (cell number), respectively (Figure 2A,C). In contrast, a significant increase in HCAEC metabolic activity by 20% to 30% was registered in presence of 6 µM and 10 µM CBD (Figure 2A), whereas 10 µM CBD on the other hand reduced the number of HCAEC by about 30% (Figure 2C). Accordingly, as with HCASMC, 10 µM CBD was not included in further experiments. Treatment with THCV up to 10 µM had no effect on HCAEC metabolic activity and cell number (Figure 2B,D). For comparison, the mTOR inhibitor sirolimus, traditionally used in DES, was also investigated. In contrast to the cannabinoids, sirolimus caused a significant reduction in metabolic activity and cell number even at low subnanomolar concentrations (Supplementary Figure S1).

### 3.3. CBD and THCV Elicit Concentration-Dependent Inhibition of IL-1β- and LPS-Induced VCAM-1 but Not ICAM-1 mRNA Levels in HCAEC

During inflammation, the recruitment of immunocompetent cells such as leukocytes plays an important role. Accordingly, proinflammatory mediators such as chemokines and adhesion molecules are highly expressed in inflamed tissue [9,12]. 

**Figure 3:**
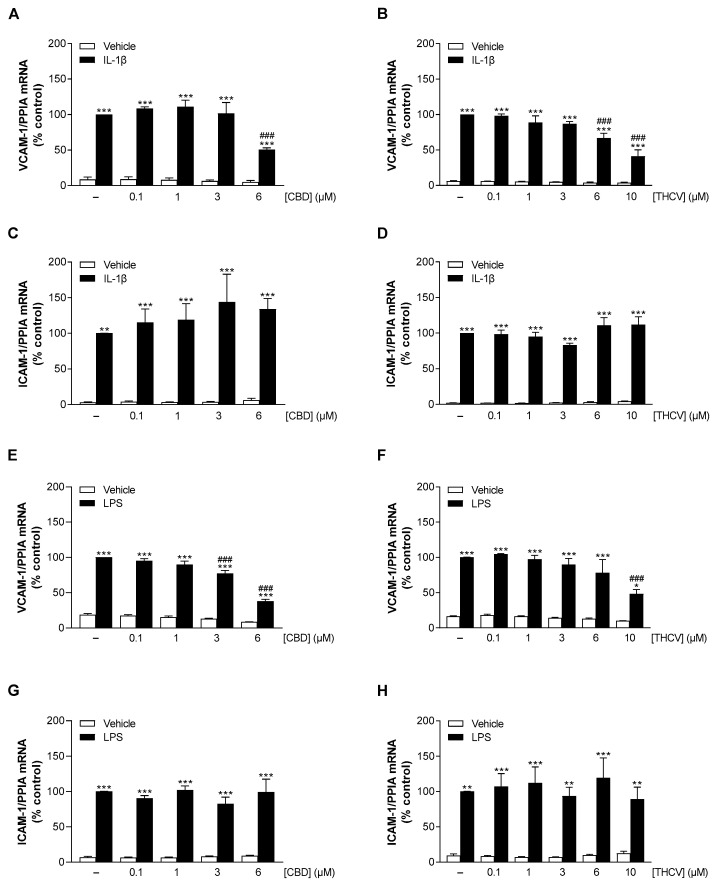
Effect of CBD and THCV on VCAM-1 and ICAM-1 mRNA expression in HCAEC under basal, IL-1β- and LPS-induced conditions. HCAEC were incubated for 24 h with 10 ng/mL IL-1β or its vehicle and increasing concentrations of CBD (**A**,**C**), THCV (**B**,**D**), or with vehicle. For LPS-stimulated cells, HCAEC were preincubated with increasing concentrations of CBD (**E**,**G**), THCV (**F**,**H**), or vehicle for 1 h and then co-incubated with 1 µg/mL LPS or its vehicle for 24 h. Thereafter, mRNA expression was determined by qRT-PCR. Cells treated with vehicle in combination with IL-1β or LPS were set 100%. Data are presented as means ± SEM of *n* = 3 (3 independent experiments, (**B**,**D**,**F**,**H**)) or *n* = 4 (4 independent experiments, (**A**,**C**,**E**,**G**)). * *p* ≤ 0.05, ** *p* ≤ 0.01, *** *p* ≤ 0.001 vs. non-stimulated vehicle control group; ### *p* ≤ 0.001 vs. IL-1β- or LPS-stimulated cells; one-way ANOVA plus Bonferroni post hoc test.

To reveal possible anti-inflammatory properties of CBD and THCV, the mRNA levels of the adhesion molecules VCAM-1 and ICAM-1 in HCAEC were examined by qRT-PCR after 24 h incubation with the respective cannabinoid. To elicit an inflammatory state in the cells, HCAEC were stimulated with IL-1β or LPS, which in both cases resulted in a significant increase in VCAM-1 and ICAM-1 mRNA expression compared with vehicle-treated cells (Figure 3A–H). CBD caused significant suppression of IL-1β- and LPS-stimulated VCAM-1 mRNA levels starting at 6 µM (Figure 3A) and 3 µM (Figure 3E), respectively. On the other hand, ICAM-1 mRNA levels were not subject to inhibitory regulation by CBD under either IL-1β or LPS stimulation (Figure 3C,G). THCV showed concentration-dependent attenuation of VCAM-1 mRNA expression in both IL-1β- and LPS-stimulated HCAEC with significant effects starting at 6 µM (Figure 3B) and 10 µM (Figure 3F), respectively. Consistent with the CBD data, THCV also did not alter ICAM-1 mRNA expression under IL-1β or LPS stimulation (Figure 3D,H).

### 3.4. CBD and THCV Cause a Concentration-Dependent Decrease in VCAM-1 but Not ICAM-1 Protein Levels in HCAEC under IL-1β- and LPS-Induced Conditions

Next, the effect of CBD and THCV on protein expression of the adhesion molecules VCAM-1 and ICAM-1 in IL-1β- and LPS-stimulated HCAEC after 24 h of treatment was examined by Western blot analysis. In addition, protein expression was analyzed under basal conditions.

**Figure 4:**
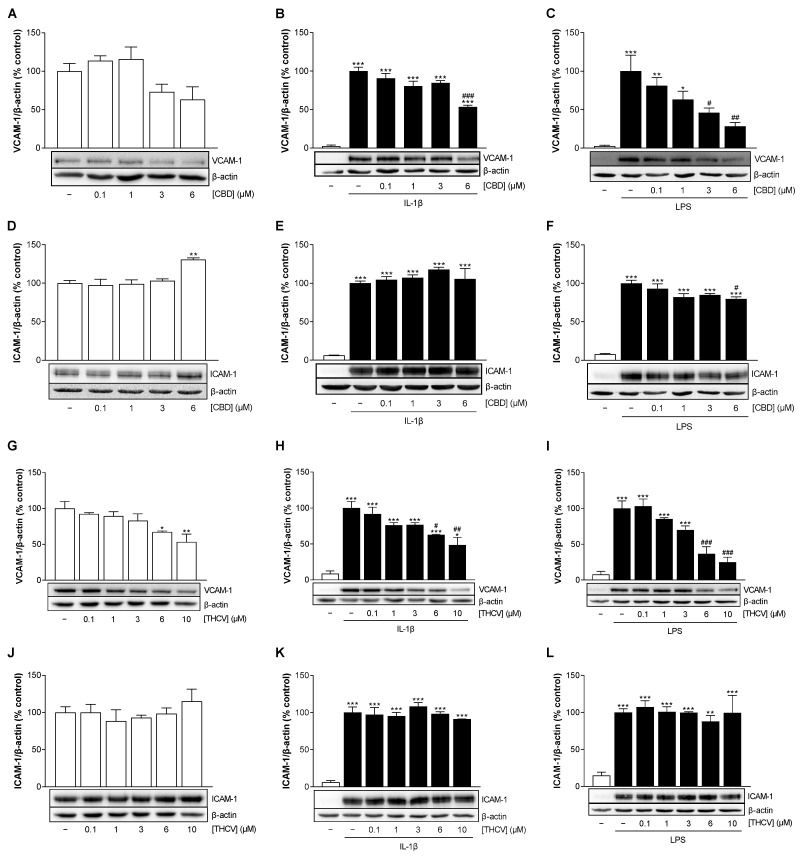
Effect of CBD and THCV on VCAM-1 and ICAM-1 protein expression in HCAEC under basal, IL-1β-, and LPS-induced conditions. For analysis under basal conditions, HCAEC were incubated with increasing concentrations of CBD (**A**,**D**), THCV (**G**,**J**), or vehicle control for 24 h. For analysis under IL-1β-induced conditions, HCAEC were incubated with 10 ng/mL IL-1β or its vehicle and increasing concentrations of CBD (**B**,**E**) or THCV (**H**,**K**) or its vehicle for 24 h. For analysis under LPS-induced conditions, HCAEC were preincubated with increasing concentrations of CBD (**C**,**F**) or THCV (**I**,**L**) or its vehicle for 1 h, followed by the addition of 1 µg/mL LPS or its vehicle and further incubation for 24 h. Protein expression was determined by Western blot analysis, with representative blots shown here. In (**C**,**F**), in (**G**,**J**), as well as in (**I**,**L**), the same β-actin blots are shown, since the proteins analyzed in these Western blots were separated on the same gel. Cells treated with vehicle (**A**,**D**,**G**,**J**) or vehicle in combination with IL-1β (**B**,**E**,**H**,**K**) or LPS (**C**,**F**,**I**,**L**) served as control (100%). Data are expressed as means ± SEM of *n* = 3 (3 independent experiments, (**A**–**C**,**E**–**L**)) or *n* = 4 (4 independent experiments, **D**). * *p* ≤ 0.05, ** *p* ≤ 0.01, *** *p* ≤ 0.001 vs. corresponding control; # *p* ≤ 0.05, ## *p* ≤ 0.01, ### *p* ≤ 0.001 vs. IL-1β- or LPS-stimulated cells; one-way ANOVA plus Bonferroni post hoc test.

Examination of VCAM-1 and ICAM-1 protein expression under basal conditions revealed a decrease in VCAM-1 protein levels mediated by CBD (Figure 4A, not significant) and THCV (Figure 4G) and an increase in ICAM-1 protein levels at the highest CBD (Figure 4D) and THCV concentrations (Figure 4J, not significant).

Stimulation of HCAEC by IL-1β and LPS significantly upregulated VCAM-1 and ICAM-1 expression compared with vehicle-treated cells (Figure 4B,C,E,F,H,I,K,L). Here, both CBD and THCV caused a concentration-dependent downregulation of VCAM-1 protein expression in IL-1β- (Figure 4B,H) as well as LPS-stimulated HCAEC (Figure 4C,I). In the same samples, ICAM-1 protein expression under IL-1β stimulation (Figure 4E,K) was not affected by either CBD or THCV (Figure 4E,K), whereas ICAM-1 levels under LPS-induced conditions showed a slight decrease in the presence of CBD but not THCV (Figure 4F,L). 

### 3.5. CBD and THCV Reduce VCAM-1 Protein Levels in HCAEC Independently of Cannabinoid Receptors CB_1_ and CB_2_, and TRPV1

**Figure 5:**
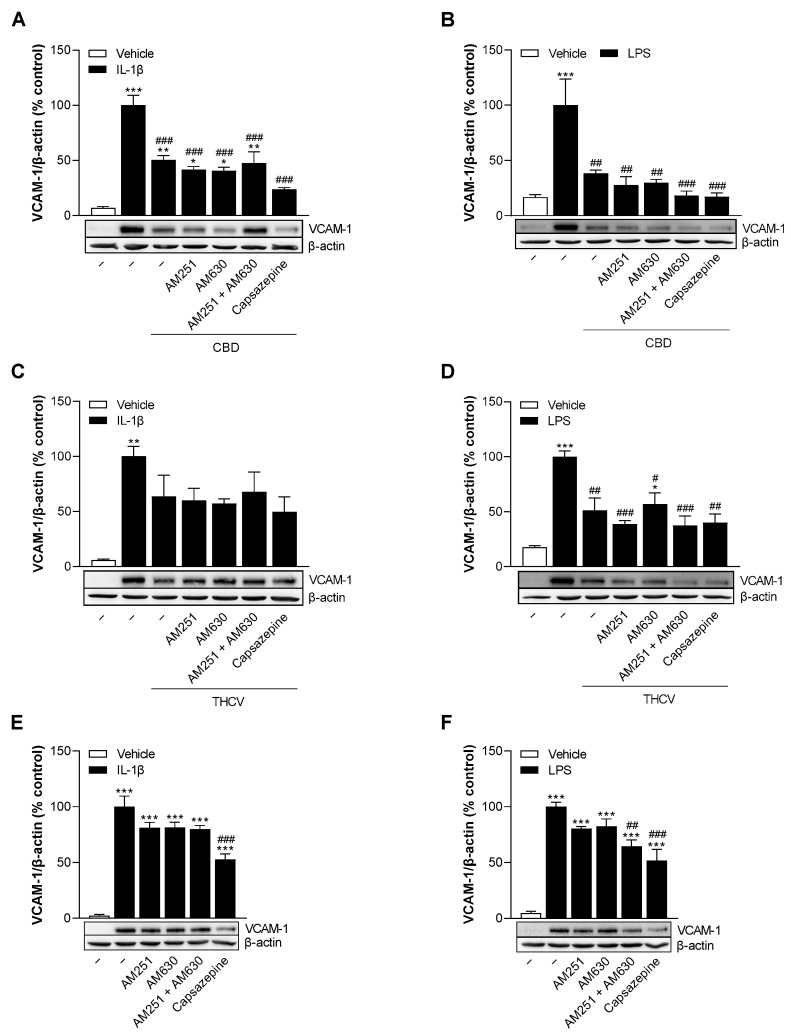
Effect of antagonists against cannabinoid receptors CB_1_ and CB_2_ and TRPV1 on VCAM-1 protein levels reduced by CBD and THCV in IL-1β- and LPS-stimulated HCAEC. HCAEC were preincubated with 1 µM AM251, 1 µM AM630, and 1 µM capsazepine for 1 h followed by the addition of 10 ng/mL IL-1β, 6 µM CBD (**A**), 10 µM THCV (**C**), or vehicles and subsequent 24 h co-incubation of cells with the compounds or their vehicles. For LPS-stimulated cells, HCAEC were preincubated with receptor antagonists for 1 h and then preincubated with 6 µM CBD (**B**), 10 µM THCV (**D**), or vehicle for an additional 1 h. This was followed by the addition of 1 µg/mL LPS or its vehicle and a further 24 h co-incubation of cells with the compounds or their vehicles. As a control, the effects of the receptor antagonists were analyzed upon co-incubation with IL-1β (**E**) and LPS (**F**) for 24 h without cannabinoid addition. Thereafter, protein expression was determined by Western blot analysis. The blots shown are representative. Cells treated with vehicle in combination with IL-1β or LPS were set 100%. Data are presented as means ± SEM of *n* = 3 (3 independent experiments). * *p* ≤ 0.05, ** *p* ≤ 0.01, *** *p* ≤ 0.001 vs. non-stimulated vehicle control group (open columns); # *p* ≤ 0.05, ## *p* ≤ 0.01, ### *p* ≤ 0.001 vs. the 100% IL-1β- or LPS stimulated control group; one-way ANOVA plus Bonferroni post hoc test.

To investigate the molecular mechanism by which CBD and THCV downregulate VCAM-1 expression in HCAEC, we focused on cannabinoid receptors CB_1_ and CB_2_ and transient receptor potential vanilloid 1 (TRPV1), whose modulation seem to have a particular role in the biological effects of the phytocannabinoids studied [27,28,29,30]. Therefore, cells were pre-incubated with the cannabinoid receptor antagonists AM251 (CB_1_), AM630 (CB_2_) and capsazepine (TRPV1) prior to treatment with 6 µM CBD or 10 µM THCV and stimulation with IL-1β or LPS. Initially, all receptor antagonists alone showed no toxic effects on HCAEC as measured by colorimetric WST-1 determination and crystal violet staining after 24 h (Supplementary Table S1). As in previous experiments, stimulation of HCAEC with IL-1β and LPS significantly upregulated VCAM-1 expression compared with vehicle-treated cells (Figure 5). The downregulation of VCAM-1 by CBD and THCV was not prevented by either CB_1_, CB_2_, or TRPV1 receptor antagonists (Figure 5A–D). Furthermore, the influence of the receptor antagonists themselves on VCAM-1 protein expression in HCAEC was also analyzed (Figure 5E,F). Here, we noticed that capsazepine in particular showed an inhibitory effect on IL-1β- and LPS-stimulated VCAM-1 protein formation in HCAEC.

### 3.6. p38 MAPK, NF-κB, HDAC, and ROS Mediate IL-1β- and LPS-Induced VCAM-1 Expression in HCAEC, Whereas p42/44 MAPK and JNK Are Not Involved

To gain more detailed insights into the molecular mechanism of VCAM-1 downregulation by CBD and THCV, the signal transduction pathways underlying IL-1β- and LPS-induced VCAM-1 protein expression were first investigated by testing corresponding inhibitors. Here, we focused on the inhibition of p38 mitogen-activated protein kinases (MAPK), p42/44 MAPK, c-Jun N-terminal kinases (JNK), nuclear factor kappa-B (NF-κB), histone deacetylases (HDAC), and reactive oxygen species (ROS) by SB203580, PD98059, SP600125, BAY 11-7082, trichostatin A (TSA), and *N*-acetyl-L-cysteine (NAC), respectively. For this purpose, HCAEC were preincubated with the above inhibitors for 1 h, then stimulated with IL-1β or LPS for 24 h, and finally analyzed by Western blot, with ICAM-1 quantified in addition to VCAM-1 for comparison. The effects of the inhibitors on HCAEC viability, as measured by the WST-1 colorimetric assay and crystal violet staining, are summarized in Supplementary Tables S2–S4. 

**Figure 6:**
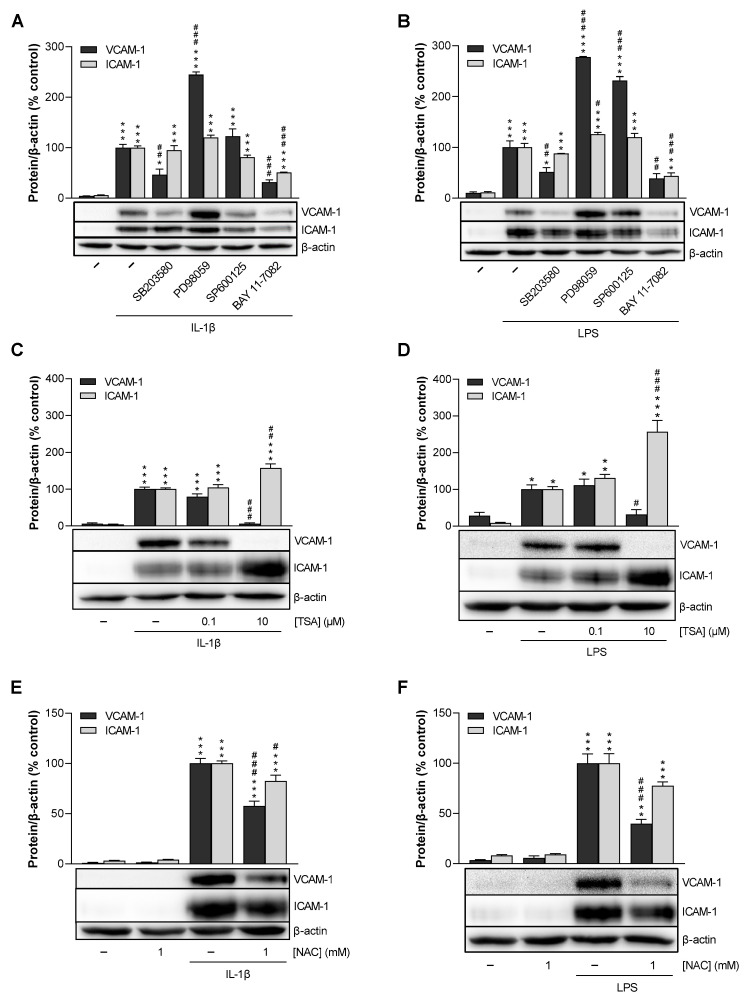
Effect of MAPK, NF-κB (**A**,**B**), HDAC (**C**,**D**) and ROS inhibitors (**E**,**F**) on IL-1β- and LPS-induced VCAM-1 and ICAM-1 protein expression in HCAEC. HCAEC were preincubated with the respective inhibitor (SB203580, PD98059, SP600125: 10 µM; BAY 11-7082: 1 µM; TSA: 0.1 µM, 10 µM; NAC: 1 mM) or vehicle for 1 h, followed by the addition of 10 ng/mL IL-1β (**A**,**C**,**E**) or 1 µg/mL LPS (**B**,**D**,**F**) or vehicle and further co-incubation of the substances for 24 h. Protein expression was then determined by Western blot analysis. The blots shown are representative. Cells treated with inhibitor vehicle and IL-1β or LPS served as controls (set as 100%). Data are shown as means ± SEM of *n* = 3 (3 independent experiments). * *p* ≤ 0.05, ** *p* ≤ 0.01, *** *p* ≤ 0.001 vs. non-stimulated vehicle control group; # *p* ≤ 0.05, ## *p* ≤ 0.01, ### *p* ≤ 0.001 vs. the 100% IL-1β- or LPS stimulated control group; one-way ANOVA plus Bonferroni post hoc test.

Inhibition of p38 MAPK resulted in a decrease of VCAM-1 expression in stimulated HCAEC by approximately 50%, whereas ICAM-1 expression remained unchanged, similar to CBD and THCV (Figure 6A,B). The NF-κB inhibitor BAY 11-7082 showed equally inhibitory effects on both adhesion molecules (Figure 6A,B). On the other hand, inhibition of p42/44 MAPK and JNK resulted in an increase in VCAM-1 protein expression in LPS-stimulated HCAEC (Figure 6B). In contrast, in IL-1β-stimulated cells, only PD98059 increased VCAM-1 expression, whereas SP600125 did not alter VCAM-1 or ICAM-1 levels (Figure 6A). As shown in Figure 6C,D, the HDAC inhibitor TSA at a concentration of 10 µM significantly decreased VCAM-1 expression while increasing ICAM-1 expression. At the same time, a TSA concentration of 0.1 µM did not regulate either adhesion molecule. TSA showed viability- or cell number-reducing effects at concentrations of 1 µM and above (Supplementary Table S4), consistent with its recently demonstrated antiproliferative effect on HUVEC [31]. Remarkably, however, the viability reduction observed at a TSA concentration of 10 µM (Supplementary Table S4) was accompanied by a concomitant much greater down-regulation of VCAM-1. Finally, scavenging of ROS by NAC resulted in a marked and highly significant decrease in IL-1β- and LPS-induced VCAM-1 expression with comparatively only weak inhibitory effects on ICAM-1 expression (Figure 6E,F). 

### 3.7. CBD and THCV Do Not Cause Inhibition of IL-1β- and LPS-Induced Activation of p38 MAPK

**Figure 7:**
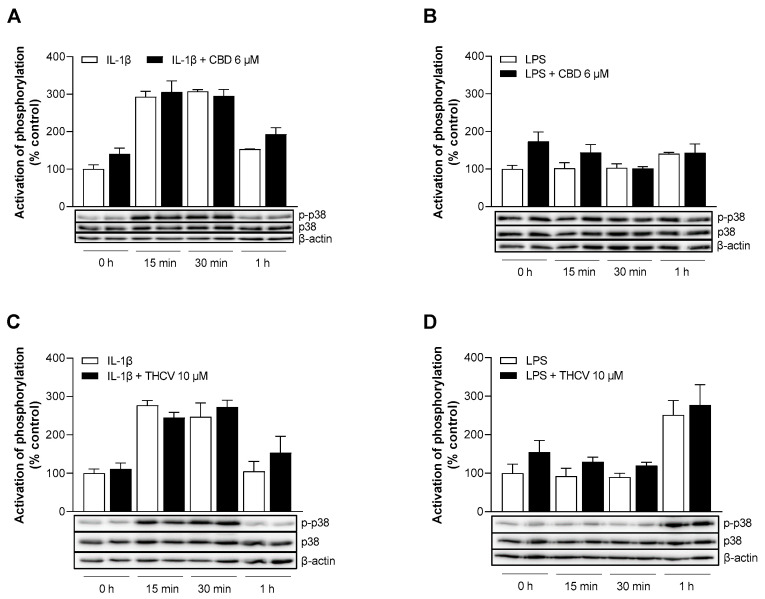
Effect of CBD and THCV on the phosphorylation of p38 MAPK in IL-1β- and LPS-stimulated HCAEC. HCAEC were incubated for the indicated times with 10 ng/mL IL-1β in the presence of 6 µM CBD (**A**), 10 µM THCV (**C**), or vehicle. For LPS-stimulated cells, HCAEC were preincubated with 6 µM CBD (**B**), 10 µM THCV (**D**), or vehicle for 1 h and then co-incubated with 1 µg/mL LPS for the indicated time points. At the 0 h time point, the HCAEC were treated with the test substances, and the supernatants were then immediately collected and the cells processed for subsequent analysis. Phosphorylation of proteins was then determined by Western blot analysis. The blots shown are representative. Cells treated with IL-1β or LPS for 0 h were used as controls (100%). Data are shown as means ± SEM of *n* = 3 (3 independent experiments). Statistical significance between the two groups of an incubation period was excluded using Student’s unpaired two-tailed *t* test.

A well-known signal transduction in response to stress stimuli such as IL-1β or LPS is the p38 MAPK signaling pathway [32,33]. To analyze the involvement of p38 MAPK in CBD- and THCV-mediated effects on VCAM-1 downregulation, the activation of p38 MAPK was monitored by comparing the phosphorylated form of p38 MAPK with unphosphorylated p38 MAPK after 0 min, 15 min, 30 min, and 1 h of incubation. For this purpose, HCAEC were activated with IL-1β or LPS and treated with 6 µM CBD or 10 µM THCV, and protein phosphorylation was detected by Western blot analysis. Activation of p38 MAPK phosphorylation could be registered after 15 and 30 min of IL-1β stimulation and reached here up to approximately three-fold increases compared with the zero time point, then returning to the approximate baseline level after 1 h incubation (Figure 7A,C). However, neither CBD nor THCV were able to inhibit IL-1β-activated phosphorylation (Figure 7A,C). Moreover, under LPS-induced conditions, no inhibitory interference of CBD and THCV with the corresponding LPS controls was registered. In contrast to IL-1β, a different stimulation pattern was evident in the presence of LPS, with comparatively late 1.2-fold (Figure 7B) and 2.5-fold (Figure 7D) activations after 1 h, respectively.

### 3.8. CBD and THCV Inhibit LPS-, but Not IL-1β-Induced Activation of NF-κB in HCAEC 

**Figure 8:**
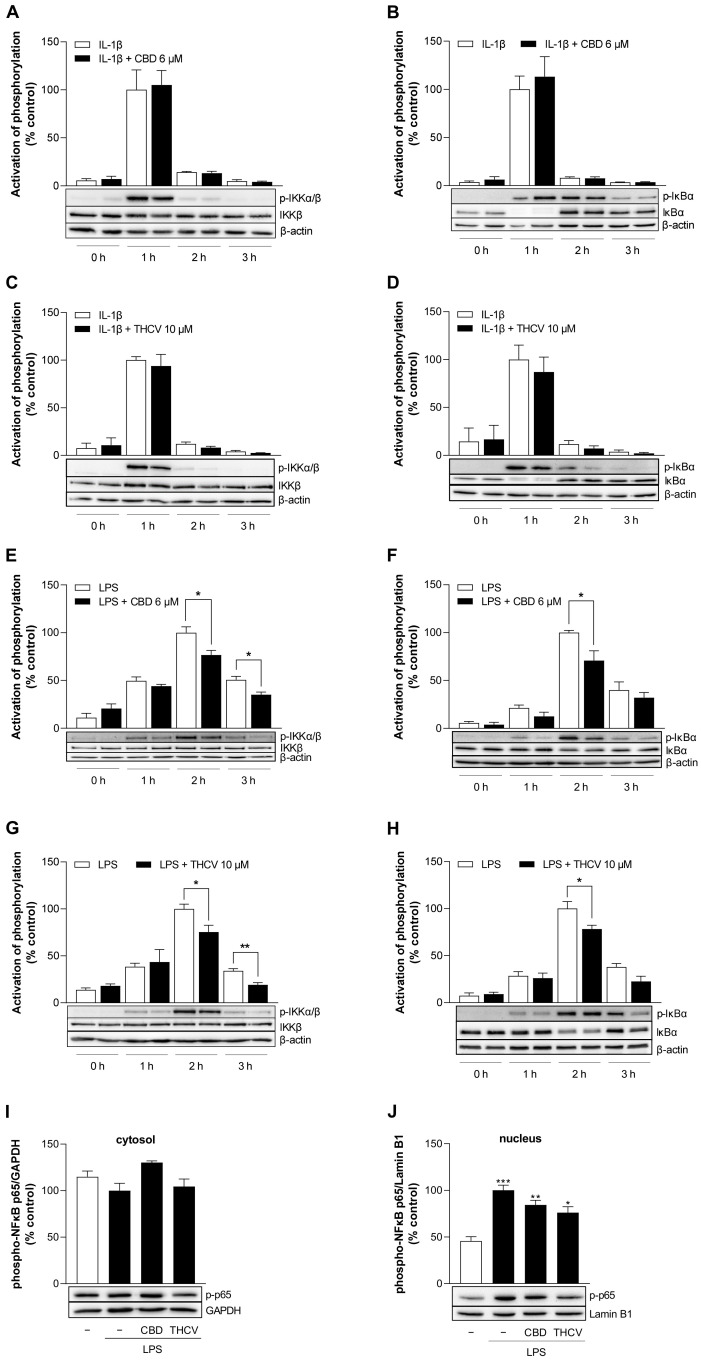
Effect of CBD and THCV on regulators of the NF-κB signaling pathway in IL-1β- and LPS-stimulated HCAEC. HCAEC were incubated with 10 ng/mL IL-1β and 6 µM CBD (**A**,**B**), 10 µM THCV (**C**,**D**), or vehicle for the indicated times. For LPS-stimulated cells, HCAEC were preincubated with 6 µM CBD (**E**,**F**), 10 µM THCV (**G**,**H**), or vehicle for 1 h and co-incubated with the appropriate compounds after the addition of 1 µg/mL LPS for the indicated times. For analysis of phospho-p65 NF-κB in the cytosolic (**I**) and nuclear fractions (**J**), HCAEC were preincubated with 6 µM CBD, 10 µM THCV, or vehicle for 1 h, followed by the addition of 1 µg/mL LPS and co-incubation with the compounds for 2 h. The blots shown are representative. At the 0 h time point, the HCAEC were treated with the test substances, and the supernatants were then immediately collected and the cells processed for subsequent Western blot analysis. Cells treated with IL-1β for 1 h or with LPS for 2 h were used as controls (100%). Data are presented as means ± SEM of *n* = 3 (3 independent experiments, (**A**–**D**,**F**,**I**,**J**)) or *n* = 4 (4 independent experiments, (**E**,**G**,**H**)). * *p* ≤ 0.05, ** *p* ≤ 0.01 vs. corresponding vehicle control; Student’s unpaired two-tailed *t* test (**A**–**H**). * *p* ≤ 0.05, ** *p* ≤ 0.01, *** *p* ≤ 0.001 vs. IL-1β- or LPS-stimulated cells; one-way ANOVA plus Bonferroni post hoc test (**I**,**J**).

The NF-κB signaling pathway plays an important role in the regulation of the immune response [34,35]. To analyze the involvement of the canonical NF-κB pathway in the CBD- and THCV-induced effects on VCAM-1 protein levels, NF-κB activation was monitored by examining the phosphorylation of NF-κB kinase subunit inhibitor beta (IKKβ) and NF-κB inhibitor alpha (IκBα). For this purpose, HCAEC were activated with IL-1β or LPS and treated with 6 µM CBD and 10 µM THCV for 0 h, 1 h, 2 h, and 3 h, respectively. LPS-stimulated cells were preincubated with phytocannabinoids for 1 h. Subsequently, protein phosphorylation was detected by Western blot analysis. Here, IL-1β caused a maximum of IKKβ and IκBα phosphorylation after 1 h (Figure 8A–D), whereas LPS led to a maximal activation after 2 h (Figure 8E–H). In IL-1β-stimulated HCAEC, neither CBD nor THCV was able to decrease IKKβ or IκBα phosphorylation (Figure 8A–D). On the other hand, treatment with both phytocannabinoids caused a reduction in phospho-IKKβ after 2 h and 3 h of LPS stimulation (Figure 8E,G). In this regard, CBD and THCV significantly reduced phosphorylation by up to 25%. Similarly, CBD and THCV reduced IκBα phosphorylation by 30% and 20%, respectively, in LPS-stimulated HCAEC after 2 h (Figure 8F,H). 

In support of these results, translocation of the phosphorylated form of transcription factor p65 (RelA) from the cytosol to the nucleus was examined under LPS-stimulated conditions after 2 h (Figure 8I,J). This showed that LPS-induced translocation of phospho-p65 to the nucleus was reduced from 100% to about 85% by CBD and to about 75% by THCV, although no significance was registered in either case (Figure 8J). Phospho-p65 concentration in the cytosol showed no significant change (Figure 8I).

### 3.9. CBD Induces Protein Expression of HDAC5 under Stimulated and Unstimulated Conditions in HCAEC

**Figure 9:**
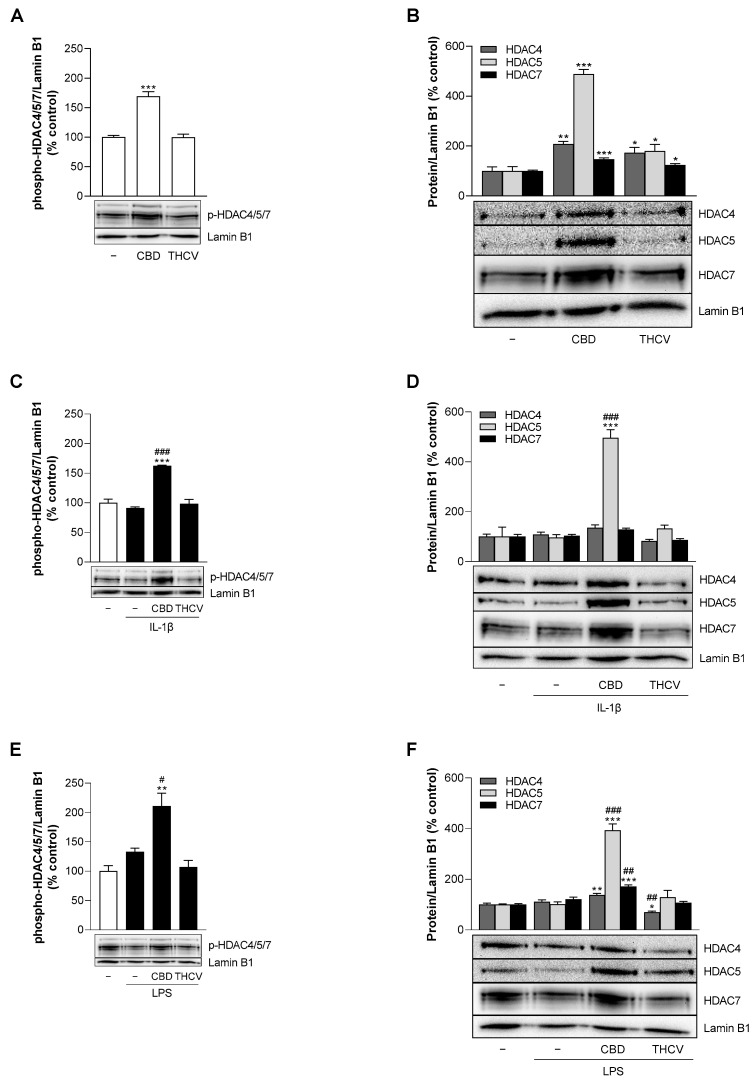
Effect of CBD and THCV on phosphorylation of class II HDAC in HCAEC under basal conditions or under IL-1β or LPS stimulation. For analysis under basal conditions, HCAEC were incubated with 6 µM CBD, 10 µM THCV, or vehicle for 24 h (**A**,**B**). For analysis under IL-1β-induced conditions, HCAEC were incubated with 10 ng/mL IL-1β together with 6 µM CBD, 10 µM THCV, or vehicle control for 24 h (**C**,**D**). For analysis under LPS-induced conditions, HCAEC were preincubated with 6 µM CBD, 10 µM THCV, or vehicle for 1 h, followed by the addition of 1 µg/mL LPS or its vehicle and further incubation for 24 h (**E**,**F**). Thereafter, protein expression was determined by Western blot analysis. The blots shown are representative. Vehicle-treated cells were used as controls (100%). Data are shown as means ± SEM of *n* = 3 (3 independent experiments, (**C**–**F**)) or *n* = 4 (4 independent experiments, (**A**,**B**)). * *p* ≤ 0.05, ** *p* ≤ 0.01, *** *p* ≤ 0.001 vs. corresponding vehicle control; # *p* ≤ 0.05, ## *p* ≤ 0.01, ### *p* ≤ 0.001 vs. IL-1β- or LPS-stimulated cells; one-way ANOVA plus Dunnett (**A**,**B**) or Bonferroni post hoc test (**C**–**F**).

Several HDAC enzymes have been identified as mediators of cytokine-induced VCAM-1 expression [36,37,38,39]. As shown before, the pan-HDAC inhibitor TSA causes a significant decrease in VCAM-1 protein expression under stimulated conditions in HCAEC (Figure 6C,D). To investigate HDAC regulation in our experimental setup, the phosphorylation of class II HDAC4, 5, and 7 at 24 h was examined by Western blot analysis. Under basal conditions, CBD significantly increased phospho-HDAC4/5/7 (Figure 9A) and the unphosphorylated forms of HDAC4, 5, and 7, with the strongest effect observed for HDAC5 (Figure 9B). Significant but comparatively weaker increases in HDAC4, 5, and 7 were also registered in the presence of THCV (Figure 9B). IL-1β and LPS stimulation itself had no significant effect on phospho-HDAC4/5/7 in HCAEC, as well as on the unphosphorylated forms examined (Figure 9C–F). Consistent with the above studies under basal conditions, the phosphorylated forms of HDAC4/5/7 were also strongly upregulated by CBD, but not by THCV, under IL-1β and LPS stimulation (Figure 9C,E). As for the unphosphorylated forms, CBD showed a stimulatory effect on the formation of HDAC5 under IL-1β-induced (Figure 9D) and on HDAC4, 5, and 7 under LPS-induced conditions (Figure 9F). Of these, the expression of HDAC5 was also most strongly upregulated by CBD under LPS stimulation (Figure 9F).

### 3.10. CBD and THCV Do Not Cause Significant Inhibition of IL-1β- and LPS-Induced Monocyte Adhesion to HCAEC

**Figure 10:**
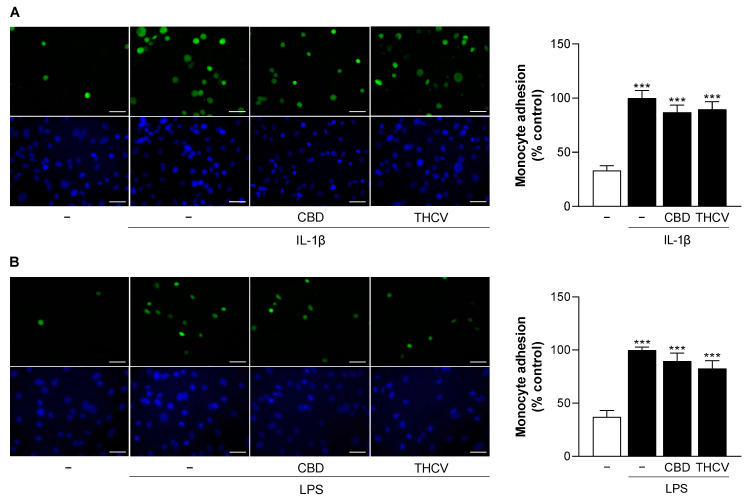
Effect of CBD and THCV on adhesion of THP-1 monocytes to IL-1β- and LPS-stimulated HCAEC. HCAEC were incubated for 24 h with 10 ng/mL IL-1β or its vehicle and 6 µM CBD, 10 µM THCV or its vehicle (**A**). For LPS-stimulated cells, HCAEC were preincubated with 6 µM CBD or 10 µM THCV or vehicle for 1 h, followed by the addition of 1 µg/mL LPS or its vehicle and further co-incubation with the compounds or vehicles for 24 h (**B**). Calcein-AM-labeled THP-1 cells (green) were then attached to endothelial cells for 30 min before analysis by fluorescence microscopy. The nuclei of all cells are shown in blue. The images shown are representative (scale bar, 50 µm). Cells treated with IL-1β or LPS were used as controls (100%). Data are presented as means ± SEM of *n* = 7–8 (4 independent experiments). *** *p* ≤ 0.001 vs. vehicle control; one-way ANOVA plus Bonferroni post hoc test.

Monocyte recruitment and infiltration into inflamed tissue is a crucial step in atherosclerosis, formation of neointimal hyperplasia, and subsequent restenosis [12,40]. In this context, downregulation of the adhesion molecule VCAM-1, as in the present study, suggests a possible disruption of monocyte adhesion. Consistently, therefore, the adhesion of monocytes to activated endothelial cells was studied. To this end, HCAEC were stimulated with IL-1β and LPS in the presence or absence of 6 µM CBD or 10 µM THCV for 24 h, followed by adherence of labeled human monocytic cells (THP-1) to HCAEC during a 30-min incubation period and subsequent fluorescence microscopy analysis. Activation of HCAEC by IL-1β and LPS resulted in a two-fold and three-fold increase in monocyte adhesion, respectively, compared with non-activated cells (Figure 10). Both CBD and THCV showed a slight but non-significant downregulation of monocyte adhesion in IL-1β- (Figure 10A) and LPS-stimulated HCAEC (Figure 10B). 

## 4. Discussion

The treatment of atherosclerosis and restenosis after coronary stent implantation remains a major pharmacotherapeutic challenge requiring appropriate agents for stent coating. In the present in vitro study, non-psychoactive phytocannabinoids CBD and THCV were investigated in the two atherosclerosis-associated cell types HCASMC and HCAEC. Here, we provide first insight into the effect of CBD and THCV on PDGF-induced viability, proliferation, and migration in HCASMC and on IL-1β- and LPS-induced upregulation of mRNA as well as protein expression of adhesion molecules VCAM-1 and ICAM-1 in HCAEC and the signal transducers involved in this process.

VSMC play an important role in all stages of atherosclerosis as well as in restenosis after coronary stenting due to neointimal hyperplasia (for review, see [6,7,8]). Vascular injury affects VSMC plasticity by switching from a contractile to a synthetic/proliferative phenotype [6]. In this context, we demonstrated that PDGF significantly increases HCASMC proliferation and migration, which has previously also been shown for this growth factor in other VSMC [18,41]. However, for the first time, we were able to prove that CBD inhibits proliferation and migration of PDGF-stimulated HCASMC without concomitant impairment of cell viability. In agreement with this, a proliferation and migration inhibitory effect of CBD has been previously described in human umbilical artery smooth muscle cells [18]. Moreover, CBD has been shown to attenuate pulmonary arterial hypertension by inhibiting the proliferation of pulmonary artery smooth muscle cells and by reducing inflammation and ROS levels in vivo [42]. Thus, CBD appears to be a promising agent for preventing neointima formation associated with restenosis due to its beneficial effects in HCASMC. Furthermore, no cytotoxic effects were observed in our initial investigation of THCV in VSMC. Thereby, THCV was found to prevent PDGF-induced HCASMC migration but not proliferation, with the reasons for this differential regulation to be elucidated in future work.

In the vessel wall, coronary stenting leads to disruption of the vascular endothelium and subsequent stimulation of thrombotic and immune responses [9]. The damaged endothelium contributes to excessive proliferation and migration of VSMC (neointimal hyperplasia), which are the most common causes of in-stent restenosis. In addition, most applied proliferation-inhibiting agents not only target VSMC but also vascular endothelial cells, leading to impaired reendothelialization and wound repair, ultimately causing in-stent restenosis [9]. In the present study, CBD and THCV had no significant effect on the metabolic activity and cell number of HCAEC at concentrations up to 6 µM (CBD) and 10 µM (THCV), respectively. In contrast, at higher concentrations (10 µM), CBD significantly decreased the number of HCAEC as an indicator of cytotoxicity. Consistent with this, a previous work by our group demonstrated that lower CBD concentrations (up to 6 µM) prevented apoptotic effects on human umbilical vein endothelial cells (HUVEC) via upregulation of the enzyme heme oxygenase-1 (HO-1) and initiation of HO-1-dependent cytoprotective proautophagic processes. However, this compensation no longer functions when higher concentrations of CBD (10 µM) were used, resulting in apoptotic cell death [43]. Therefore, even in the case of non-psychoactive phytocannabinoids, it is important to ensure that concentrations or dosages are used that preserve the integrity of the vascular endothelium and allow vascular healing to occur.

When investigating the anti-inflammatory properties of potential agents for DES, leukocyte-endothelial cell interactions via the adhesion molecules VCAM-1 and ICAM-1 are of great interest [44]. Here, we demonstrate a partial anti-inflammatory effect of CBD and THCV in IL-1β- and LPS-stimulated HCAEC through concentration-dependent downregulation of VCAM-1 mRNA and protein levels, but not ICAM-1. Differential regulation of adhesion molecules is not uncommon. Similar results were obtained in tumor necrosis factor (TNF)-α-activated HUVEC upon treatment with the active aglycone genipin [45] and the diterpene tanshinone IIA [46] by upregulation of peroxisome proliferator-activated receptor (PPAR)-γ and inhibition of transcription factors GATA-6 and interferon regulatory factor (IRF)-1. In contrast, Rajesh et al. showed a decrease in VCAM-1 and ICAM-1 levels in glucose-stimulated HCAEC by CBD [19]. Our results suggest different regulatory mechanisms for IL-1β- and LPS-induced VCAM-1 and ICAM-1 expression, which need further investigation.

The initial focus of elucidating the corresponding signaling pathways of CBD- and THCV-mediated VCAM-1 downregulation was on the receptors involved in this process. Here, approaches with antagonists targeting the classical cannabinoid receptors CB_1_ and CB_2_ and TRPV1 showed no effect on CBD- and THCV-dependent downregulation of IL-1β- or LPS-induced VCAM-1 protein expression in HCAEC. In fact, CBD, which has low affinity for CB_1_ and CB_2_ receptors [28], has been repeatedly demonstrated to exert anti-inflammatory properties through cannabinoid receptor-independent mechanisms [19,25,43,47]. However, CBD-mediated vasorelaxation in human mesenteric arteries has been described as CB_1_ receptor-dependent [20]. Moreover, agonistic effects on TRPV1 have been reported for both CBD and THCV [30]. Compared to CBD, available data on the effect of THCV on CB receptors are contradictory (for review see [28]). Accordingly, THCV antagonizes cannabinoid receptor agonists in CB_1_-expressing tissues in a tissue- and ligand-dependent manner and interacts with CB_1_ receptors in vivo, behaving here either as a CB_1_ antagonist or, at higher doses, as a CB_1_ receptor agonist. On the basis of our data, we obviously cannot exclude the possibility that nonclassical cannabinoid receptors are involved in the reduction of VCAM-1 levels by CBD and THCV. In the case of CBD, for instance, other receptors involved in CBD action, such as TRPV2 [48], adenosine A_2A_ receptor [49] or GPR55 [50], could be addressed in future studies. 

To first gain insight into the regulatory mechanisms of IL-1β- and LPS-induced VCAM-1 and ICAM-1 expression, different signal transduction pathways were investigated in inhibitor experiments. Since oxidative stress is a major mediator of atherosclerosis [51], initial approaches were performed with the ROS scavenger NAC. Indeed, NAC caused a profound decrease in VCAM-1 protein levels, suggesting the involvement of ROS in IL-1β- and LPS-stimulated VCAM-1 expression in HCAEC. In parallel, comparatively lower ICAM-1 inhibition by NAC was registered. In several studies, NAC has been shown to exert anti-inflammatory properties, for instance by inhibiting inflammasome activation and inflammatory cytokine maturation in nicotine-treated human aortic endothelial cells (HAEC) [52]. Regarding its effects on adhesion molecules, NAC revealed to inhibit VCAM-1 and ICAM-1 expression in hydrogen peroxide-stimulated HUVEC [53] and visfatin-treated human microvascular endothelial cells (HMEC) [54], underscoring that endothelial dysfunction is associated with oxidative stress. Moreover, NAC has been shown to significantly abrogate IL-1β-induced VCAM-1 expression in human tracheal smooth muscle cells [37]. Thereby, IL-1β-induced VCAM-1 expression was mediated via NADPH oxidase activation dependent on MyD88, an adaptor protein involved in IL-1 signaling, and subsequent ROS formation [37].

Another known signal transduction pathway in response to stress stimuli such as IL-1β or LPS is the p38 MAPK pathway [32,33]. Here, we demonstrated that the p38 MAPK inhibitor SB203580 significantly attenuated IL-1β- and LPS-induced VCAM-1 but not concomitant ICAM-1 expression. On the other hand, virtually no inhibition of IL-1β- or LPS-induced p38 MAPK activation by CBD and THCV was detected, largely excluding p38 MAPK as a target of VCAM-1 downregulation by either phytocannabinoid. In contrast, CBD was able to attenuate diabetes-induced p38 MAPK activation in myocardial tissue [22] and hepatic ischemia-reperfusion injury in vivo [25]. In the case of another cytokine, previous reports have shown that p38 MAPK activity was not required for TNF-α-induced expression of adhesion molecules such as VCAM-1 and ICAM-1 in HUVEC, while activation of the NF-κB pathway was essential [55].

A particular focus has been directed to the NF-κB signaling pathway, which is triggered by external stressors, infectious agents, or elevated signaling molecules and controls the gene expression of various mediators leading to immune responses, cell adhesion, differentiation, or apoptosis [34]. To achieve inhibition of NF-κB, BAY 11-7082 was used in the present study, which irreversibly inhibits the IKK complex and, thus, the phosphorylation of IκBα and the translocation of NF-κB transcription factors p65/p50 from the cytosol to the nucleus [56,57]. Here, BAY 11-7082 resulted in inhibition of IL-1β- and LPS-induced VCAM-1 and ICAM-1 expression in HCAEC. On this basis, the activation cascade of the transcription factor NF-κB was examined and found that LPS activation of the NF-κB pathway was inhibited by CBD and THCV, whereas activation by IL-1β was unaffected. Thus, in LPS-stimulated HCAEC, CBD and THCV inhibit phosphorylation and consequent activation of IKKβ, phosphorylation and, hence, degradation of IκBα, and thereafter translocation of phospho-p65 to the nucleus. CBD is known to affect NF-κB activity in LPS-stimulated monocytes [58] as well as in other pathophysiological models including microglial inflammation [59] and hepatic steatosis [60]. However, prior to this work, no studies had been performed with THCV examining its effect on the NF-κB pathway. 

The inhibition of LPS-, but not IL-1β-induced IKKβ and IκBα phosphorylation by CBD and THCV may be due to regulatory mechanisms upstream of the IKK complex. For example, NF-κB activation by IL-1β is mediated by binding to IL-1 receptor types 1 and 2 [61], whereas activation by LPS is mediated by binding of Toll-like receptor 4 (TLR4) [62]. In addition, activation of TLR4 may lead to a MyD88-dependent or -independent signaling cascade, whereas activation of IL-1 receptors is MyD88-dependent [61,62]. In this context, further studies need to be conducted to investigate the regulatory mechanisms of CBD and THCV on upstream proteins of the IKK complex. As outlined above, inhibition of NF-κB by BAY 11-7082 decreased protein expression of both adhesion molecules VCAM-1 and ICAM-1, in contrast to the VCAM-1-selective effect of CBD and THCV. Again, this may indicate possible regulation of the upstream proteins of IKK by CBD and THCV. Moreover, Marui et al. [63] have shown that the VCAM-1 gene expression is more sensitive to inhibition by antioxidants and that redox mechanisms in general play an important role in the differential regulation of VCAM-1 and ICAM-1 gene expression in response to otherwise identical activating signals. Besides, oxidative stress is known to induce the NF-κB signaling pathway and NAC has been shown to inhibit the hydrogen peroxide-, TNF-α- and LPS-induced NF-κB activation [64,65,66], indicating a possible link between the antioxidant properties of CBD and THCV and their involvement in NF-κB signaling. On the other hand, NAC did not affect the IL-1β-activated NF-κB signaling pathway in macrophages [67], suggesting that additional signaling pathways may be involved in the inhibition of VCAM-1 by NAC. In support of this theory, direct antioxidant effects on redox balance as well as indirect antioxidant effects through modulation of the endocannabinoid system by CBD have been described [68]. Moreover, CBD showed antioxidant properties by reducing ROS production and phospho-p65 NF-κB expression suggesting a ROS-dependent activation of NF-κB [59]. A synthetic analogue of THCV decreased the ischemia/reperfusion injury-induced oxidative stress in vivo [26], but otherwise a link between THCV and ROS remains to be investigated.

HDAC are a class of enzymes that remove acetyl groups from an ε-*N*-acetyl-lysine amino acid on histone and non-histone proteins, thus allowing histones to coat DNA more tightly. Inhibition of HDAC accordingly leads to increased histone acetylation, which in turn results in the opening of promoter loci and facilitated interaction with transcription factors [69]. Several publications have shown involvement of HDAC in VCAM-1 expression. Thus, consistent with our findings, TSA, a selective and reversible hydroxamate inhibitor of class I and II HDAC [69], resulted in diminished VCAM-1 expression in IL-1β-stimulated human tracheal smooth muscle cells [37] as well as in HUVEC [36,70] and HAEC [71] stimulated with TNF-α. Moreover, TSA caused VCAM-1 decrease in sickle transgenic mice [72], whereas an increase in VCAM-1 expression was registered in LDL receptor-deficient mice [73]. In the HCAEC we used, the suppressive effect of HDAC inhibition was limited to VCAM-1 expression without concomitant inhibition of ICAM-1, again confirming the results of other groups obtained with HUVEC [36] and HAEC [39].

Previous analyses have registered involvement of HDAC3 [36], HDAC4 [37], HDAC5 [38], and more recently HDAC1/2 [39] in cytokine-induced VCAM-1 expression, with HDAC4 in the case of IL-1β [37]. From there, we focused on Western blot analysis of class II HDAC, which can shuttle between the nucleus and cytoplasm, acting as signal transducers [74,75]. However, no appreciable change in the expression and phosphorylation of HDAC4, 5, and 7 was detected for either IL-1β or LPS. On the other hand, an involvement of class I HDAC proteins in the inhibitory effect of TSA on VCAM-1 expression cannot be excluded. Nevertheless, the regulation of HDAC by CBD proved to be an unexpected finding in this setup. Indeed, CBD caused a significant upregulation of the phosphorylated and unphosphorylated forms of HDAC4, 5, and 7, with the strongest effect on HDAC5 expression. A possible association emerges between CBD-mediated VCAM-1 downregulation and HDAC5 phosphorylation. In another work, metformin was shown to increase phosphorylation of HDAC5, leading to export of HDAC5 from the nucleus to the cytosol and upregulation of Krüppel-like factor 2 (KLF2), and finally inhibition of VCAM-1 expression in LPS- and TNF-α-stimulated HUVEC [38]. Remarkably, in this study, LPS and TNF-α also showed no effect on phosphorylation of HDAC5 after a 16-h incubation of HUVEC, whereas a significant upregulation was observed upon concomitant and sole incubation with metformin [38]. A previous study [76] demonstrated that HDAC5 phosphorylation stimulated by fluid shear stress in HUVEC leads to nuclear export of HDAC5, dissociation of HDAC5 and myocyte enhancer factor 2 (MEF2), and, thus, enhanced transcriptional activity of MEF2. This, in turn, results in the expression of KLF2 and endothelial nitric oxide synthase (eNOS), with the NO formed in the process contributing to vascular homeostasis and having vasoprotective and anti-inflammatory effects [77]. Since our data represent HDAC5 phosphorylation only together with phosphorylation of HDAC4 and HDAC7, a possible VCAM-1 regulation by HDAC5 phosphorylation can only be assumed and remains unclear. In addition to KLF2 and MEF2, HDACs regulate multiple transcription factors [75], underscoring the complexity of this finding. Accordingly, HDAC have been found to modulate NF-κB [78] and in this way play a role in various disease conditions including atherosclerosis [79]. Specifically, HDAC4 was shown to block TNF-α- or LPS-stimulated NF-κB activation via inhibition of IκBa degradation [80]. 

The recruitment and adhesion of circulating monocytes to activated endothelium is a multistep process mediated by chemokines, monocyte integrins, and endothelial adhesion molecules [81,82]. Our study shows that stimulation with IL-1β and LPS increases the adhesion of THP-1 monocytes to HCAEC, which is most likely mediated by the demonstrated upregulation of the adhesion molecules VCAM-1 and ICAM-1. Interestingly, treatment of HCAEC with CBD and THCV was unable to block the induced adhesion of THP-1 cells to endothelial cells, although VCAM-1 expression was significantly reduced. Previous studies found that CBD reduced high glucose-induced adhesion of monocytes to the endothelium when VCAM-1 and ICAM-1 expression were simultaneously blocked [19]. Our results suggest that regulation of VCAM-1 alone, when the expression of ICAM-1 is not impaired, is not sufficient to block monocyte adhesion. In addition, other adhesion molecules such as E-selectin and P-selectin, which were not examined in the present study, also play a functionally important role in monocyte adhesion [83]. Further studies should be conducted in this regard, including also other cannabinoids.

## 5. Conclusions

**Figure 11:**
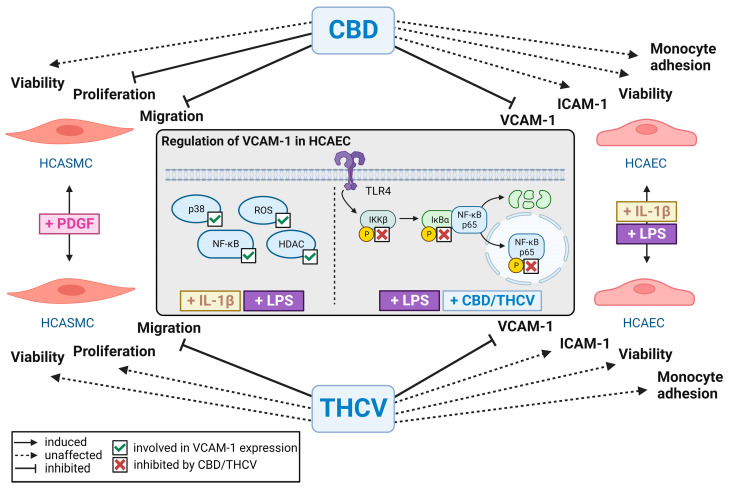
Summary of the effect of CBD and THCV on proatherosclerotic and proinflammatory properties of HCASMC and HCAEC (Created with BioRender.com, accessed on 18 August 2023). Common abbreviations used have been explained in the manuscript when first mentioned.

In summary, we found that CBD and THCV at nontoxic concentrations exerted inhibitory effects on PDGF-triggered potentially proatherosclerotic effects of HCASMC, which manifested at the level of proliferation (CBD) and migration (CBD, THCV). Furthermore, with the downregulation of IL-1β- and LPS-stimulated VCAM-1 expression by nontoxic concentrations of CBD and THCV, we were able to demonstrate a previously unknown component of the anti-inflammatory effect of both compounds on HCAEC. Corresponding effects could not be demonstrated for ICAM-1, another adhesion molecule, and were mediated independently of classical cannabinoid receptor signaling. The signaling pathways underlying IL-1β- and LPS-induced VCAM-1 expression in HCAEC appear to involve ROS, p38 MAPK, and NF-κB, with inhibition of NF-κB activation by both CBD and THCV demonstrated under LPS stimulation. The corresponding findings are summarized in Figure 11. Finally, the lack of anti-adhesive effect of CBD and THCV at the level of monocyte-endothelial interaction, despite inhibited VCAM-1 expression, suggests that additional inhibition of other adhesion molecules is required for this process. Consequently, both cannabinoids exert potentially valuable pharmacological properties in vitro with regard to antiatherogenic and anti-inflammatory effects in human coronary artery cells, which should be further investigated in future preclinical studies.

## Data Availability

Data are available upon reasonable request from the first author.

## Supplementary Materials

The following supporting information can be downloaded at: https://www.mdpi.com/article/10.3390/cells12192389/s1, Supplementary Figure S1: Effect of sirolimus on metabolic activity and cell number of HCAEC; Supplementary Table S1: Effect of cannabinoid receptor antagonists on metabolic activity and cell number of HCAEC under basal, IL-1β-, and LPS-stimulated conditions; Supplementary Table S2: Effect of MAPK inhibitors on metabolic activity and cell number of HCAEC under basal, IL-1β-, and LPS-stimulated conditions; Supplementary Table S3: Effect of NF-κB inhibitor BAY 11-7082 (BAY) on metabolic activity and cell number of HCAEC under basal, IL-1β-, and LPS-stimulated conditions; Supplementary Table S4: Effect of HDAC inhibitor trichostatin A (TSA) on metabolic activity and cell number of HCAEC under basal, IL-1β-, and LPS-stimulated conditions.
