# Supplementary material for: Non-Psychoactive Phytocannabinoids Inhibit Inflammation-Related Changes of Human Coronary Artery Smooth Muscle and Endothelial Cells
Source: Cells. 2023 Sep 30;12(19):2389. doi: 10.3390/cells12192389 (PMC10571842; doi:10.3390/cells12192389)
Supplement: Supplementary file 1 [file cells-12-02389-s001.zip › cells-2592540-supplementary.pdf]

*cells*

*Supplementary Material*

# Non-Psychoactive Phytocannabinoids Inhibit Inflammation-Related Changes of Human Coronary Artery Smooth Muscle and Endothelial Cells

**Elisa Teichmann, Elane Blessing and Burkhard Hinz ***

Institute of Pharmacology and Toxicology, Rostock University Medical Center, Schillingallee 70, 18057 Rostock, Germany; elisa.teichmann@web.de (E.T.); elane.blessing@med.uni-rostock.de (E.B.)
* Correspondence: burkhard.hinz@med.uni-rostock.de; Tel.: +49-381-494-5770

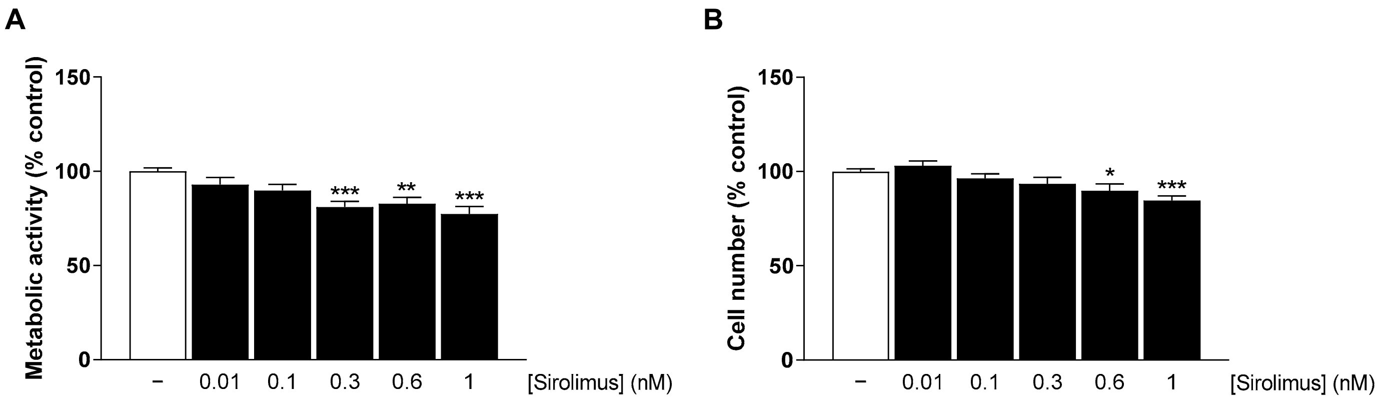


**Supplementary Figure S1:** Effect of sirolimus on metabolic activity and cell number of HCAEC. HCAEC were incubated with increasing concentrations of sirolimus or with vehicle for 24 h. Thereafter, metabolic activity was determined by WST-1 assay (**A**) and cell number by crystal violet staining (**B**). Vehicle-treated cells were used as controls (100%). Data are presented as means ± SEM of $n = 12$ (4 independent experiments). * $p \leq 0.05$, ** $p \leq 0.01$, *** $p \leq 0.001$ vs. vehicle control; one-way ANOVA plus Dunnett post hoc test.

**Supplementary Table S1:** Effect of cannabinoid receptor antagonists on metabolic activity and cell number of HCAEC under basal, IL-1β-, or LPS-stimulated conditions. HCAEC were preincubated with 1 µM AM251, 1 µM AM630, 1 µM capsazepine, or vehicle for 1 h, followed by the addition of 10 ng/mL IL-1β, 1 µg/mL LPS, or vehicle and a further 24 h incubation with the respective compounds. Subsequently, metabolic activity was determined by WST-1 assay and cell number by crystal violet staining. Cells treated with vehicle were used as control (set as 100%). Data are presented as means ± SEM of $n = 9$ (3 independent experiments). * $p \leq 0.05$ vs. vehicle control; one-way ANOVA plus Bonferroni post hoc test.

| Treatment group | Metabolic activity (%) | Cell number (%) | Treatment group | Metabolic activity (%) | Cell number (%) |
|---|---|---|---|---|---|
| Vehicle | 100.0 ± 0.9 | 100.0 ± 2.1 | Vehicle | 100.0 ± 1.9 | 100.0 ± 2.0 |
| IL-1β | 97.9 ± 2.5 | 104.2 ± 4.8 | LPS | 107.3 ± 3.5 | 106.8 ± 3.1 |
| AM251 | 107.5 ± 1.6 | 103.7 ± 1.9 | AM251 | 113.2 ± 2.3 | 103.5 ± 3.4 |
| AM251 + IL-1β | 106.2 ± 2.0 | 105.0 ± 4.1 | AM251 + LPS | 118.3 ± 5.0 | 108.5 ± 2.9 |
| AM630 | 103.1 ± 2.2 | 101.5 ± 3.1 | AM630 | 110.0 ± 2.6 | 111.5 ± 2.6 |
| AM630 + IL-1β | 94.5 ± 3.0 | 104.3 ± 4.1 | AM630 + LPS | 113.0 ± 3.9 | 111.5 ± 2.4 |
| AM251 + AM630 | 105.2 ± 1.6 | 97.0 ± 6.3 | AM251 + AM630 | 115.4 ± 4.8 | 107.2 ± 4.1 |
| AM251 + AM630 + IL-1β | 103.4 ± 3.4 | 107.3 ± 4.5 | AM251 + AM630 + LPS | 122.1 ± 6.9* | 114.3 ± 1.7 |
| Capsazepine | 108.5 ± 1.6 | 103.4 ± 5.2 | Capsazepine | 119.9 ± 8.0 | 109.1 ± 1.8 |
| Capsazepine + IL-1β | 99.1 ± 3.1 | 110.3 ± 3.8 | Capsazepine + LPS | 113.9 ± 5.4 | 107.9 ± 4.3 |

**Supplementary Table S2**: Effect of MAPK inhibitors on metabolic activity and cell number of HCAEC under basal, IL-1β-, or LPS-stimulated conditions. HCAEC were preincubated with 10 µM SB203580 (p38 MAPK inhibitor), 10 µM PD98059 (p42/44 MAPK inhibitor), 10 µM SP600125 (JNK inhibitor), or vehicle for 1 h, followed by the addition of 10 ng/mL IL-1β, 1 µg/mL LPS, or vehicle and a further 24 h incubation with the respective compounds. Subsequently, metabolic activity was determined by WST-1 assay and cell number by crystal violet staining. Cells treated with vehicle were used as control (set as 100%). Data are presented as means ± SEM of $n = 9$ (3 independent experiments). * $p \leq 0.05$ vs vehicle control; # $p \leq 0.05$ vs. stimulated control; one-way ANOVA plus Bonferroni post hoc test.

| Treatment group | Metabolic activity (%) | Cell number (%) | Treatment group | Metabolic activity (%) | Cell number (%) |
|---|---|---|---|---|---|
| Vehicle | 100.0 ± 1.5 | 100.0 ± 1.7 | Vehicle | 100.0 ± 1.3 | 100.0 ± 2.2 |
| IL-1β | 98.4 ± 3.6 | 103.2 ± 2.7 | LPS | 95.4 ± 1.4 | 98.0 ± 3.7 |
| SB203580 | 96.9 ± 1.9 | 112.4 ± 3.6 | SB203580 | 88.9 ± 2.3* | 105.3 ± 2.8 |
| SB203580 + IL-1β | 84.6 ± 3.6* | 112.1 ± 3.2 | SB203580 + LPS | 84.1 ± 1.6*# | 105.7 ± 2.8 |
| PD98059 | 87.2 ± 1.9 | 94.8 ± 2.9 | PD98059 | 74.8 ± 2.6* | 88.7 ± 1.9 |
| PD98059+ IL-1β | 80.5 ± 1.8*# | 103.5 ± 2.5 | PD98059 + LPS | 72.7 ± 2.2*# | 94.1 ± 4.5 |
| SP600125 | 80.1 ± 5.5* | 74.1 ± 4.9* | SP600125 | 67.0 ± 1.9* | 68.2 ± 2.2* |
| SP600125 + IL-1β | 89.5 ± 5.2 | 75.7 ± 2.4*# | SP600125 + LPS | 69.1 ± 1.9*# | 74.7 ± 4.7*# |

**Supplementary Table S3:** Effect of NF-κB inhibitor BAY 11-7082 (BAY) on metabolic activity and cell number of HCAEC under basal, IL-1β-, or LPS-stimulated conditions. IL-1β- and LPS-stimulated HCAEC metabolic activity and cell number. HCAEC were preincubated with increasing concentrations of BAY 11-7082 or vehicle for 1 h, followed by the addition of 10 ng/mL IL-1β, 1 μg/mL LPS, or vehicle and a further 24 h incubation with the respective compounds. Subsequently, metabolic activity was determined by WST-1 assay and cell number by crystal violet staining. Cells treated with vehicle were used as control (set as 100 %). Data are presented as means ± SEM of $n = 9$ (3 independent experiments). * $p \le 0.05$ vs. vehicle control; # $p \le 0.05$ vs. stimulated control; one-way ANOVA plus Bonferroni post hoc test.

| Treatment group | Metabolic activity (%) | Cell number (%) | Treatment group | Metabolic activity (%) | Cell number (%) |
|---|---|---|---|---|---|
| Vehicle | 100.0 ± 1.7 | 100.0 ± 3.0 | Vehicle | 100.0 ± 1.6 | 100.0 ± 1.8 |
| IL-1β | 104.2 ± 2.3 | 104.9 ± 2.6 | LPS | 99.7 ± 1.9 | 100.6 ± 2.9 |
| 0.01 μM BAY | 107.8 ±2.3 | 100.2 ± 5.3 | 0.01 μM BAY | 100.5 ± 0.9 | 111.2 ± 8.3 |
| 0.01 μM BAY + IL-1β | 110.4 ± 2.1 | 117.9 ± 5.3 | 0.01 μM BAY + LPS | 104.1 ± 1.6 | 104.3 ± 2.6 |
| 0.1 μM BAY | 116.4 ± 2.0* | 105.1 ± 3.1 | 0.1 μM BAY | 112.7 ± 2.5 | 107.8 ± 2.5 |
| 0.1 μM BAY + IL-1β | 118.9 ± 2.2* | 116.4 ± 5.2 | 0.1 μM BAY + LPS | 113.0 ± 2.7 | 105.1 ± 3.3 |
| 1 μM BAY | 132.3 ± 2.5* | 108.2 ± 5.1 | 1 μM BAY | 132.4 ± 3.8* | 105.5 ± 2.7 |
| 1 μM BAY + IL-1β | 129.8 ± 6.2*# | 114.2 ± 3.7 | 1 μM BAY + LPS | 136.9 ± 4.1*# | 108.9 ± 3.1 |
| 5 μM BAY | 25.3 ± 6.5* | 37.6 ± 6.6* | 5 μM BAY | 21.1 ± 5.9* | 42.7 ± 4.1* |
| 5 μM BAY + IL-1β | 26.0 ± 6.4*# | 40.0 ± 4.8*# | 5 μM BAY + LPS | 24.2 ± 5.4*# | 47.3 ± 5.7*# |
| 10 μM BAY | 1.7 ± 0.3* | 23.3 ± 5.5* | 10 μM BAY | 1.0 ± 0.5* | 32.9 ± 6.1* |
| 10 μM BAY + IL-1β | 1.9 ± 0.4*# | 23.0 ± 5.8*# | 10 μM BAY + LPS | 1.7 ± 0.6*# | 42.4 ± 7.5*# |

**Supplementary Table S4**: Effect of HDAC inhibitor trichostatin A (TSA) on metabolic activity and cell number of HCAEC under basal, IL-1β-, or LPS-stimulated conditions. HCAEC were preincubated with increasing concentrations of TSA or vehicle for 1 h, followed by the addition of 10 ng/mL IL-1β, 1 μg/mL LPS, or vehicle and a further 24 h incubation with the respective compounds. Subsequently, metabolic activity was determined by WST-1 assay and cell number by crystal violet staining. Cells treated with vehicle were used as control (set as 100 %). Data are presented as means ± SEM of $n = 9$ (3 independent experiments). * $p \le 0.05$ vs. vehicle control; # $p \le 0.05$ vs. stimulated control; one-way ANOVA plus Bonferroni post hoc test.

| Treatment group | Metabolic activity (%) | Cell number (%) | Treatment group | Metabolic activity (%) | Cell number (%) |
|---|---|---|---|---|---|
| Vehicle | 100.0 ± 1.8 | 100.0 ± 1.7 | Vehicle | 100.0 ± 1.4 | 100.0 ± 3.2 |
| IL-1β | 102.8 ± 2.3 | 99.6 ± 4.6 | LPS | 100.0 ± 2.2 | 105.6 ± 3.3 |
| 0.01 μM TSA | 94.5 ± 1.6 | 100.4 ± 3.1 | 0.01 μM TSA | 99.3 ± 2.6 | 107.1 ± 2.6 |
| 0.01 μM TSA + IL-1β | 102.5 ± 1.4 | 101.4 ± 3.0 | 0.01 μM TSA + LPS | 97.4 ± 2.0 | 103.0 ± 3.4 |
| 0.1 μM TSA | 94.1 ± 2.2 | 94.5 ± 2.8 | 0.1 μM TSA | 92.1 ± 1.1 | 95.9 ± 2.0 |
| 0.1 μM TSA + IL-1β | 102.1 ± 2.6 | 95.1 ± 3.0 | 0.1 μM TSA + LPS | 97.7 ± 3.0 | 99.2 ± 3.1 |
| 1 μM TSA | 74.4 ± 3.2* | 74.9 ± 2.6* | 1 μM TSA | 76.5 ± 3.4* | 77.2 ± 1.9* |
| 1 μM TSA + IL-1β | 76.2 ± 4.6*# | 71.0 ± 4.3*# | 1 μM TSA + LPS | 73.1 ± 3.2*# | 70.7 ± 1.7*# |
| 5 μM TSA | 67.6 ± 3.3* | 67.2 ± 1.8* | 5 μM TSA | 71.2 ± 3.6* | 68.8 ± 2.8* |
| 5 μM TSA + IL-1β | 62.0 ± 3.6*# | 58.4 ± 4.6*# | 5 μM TSA + LPS | 64.5 ± 4.4*# | 62.8 ± 2.5*# |
| 10 μM TSA | 67.1 ± 4.1* | 64.5 ± 1.9* | 10 μM TSA | 70.1 ± 4.3* | 72.4 ± 4.9* |
| 10 μM TSA + IL-1β | 58.3 ± 3.3*# | 57.0 ± 3.4*# | 10 μM TSA + LPS | 64.5 ± 3.8*# | 62.3 ± 3.3*# |
